# Accelerated aging with HIV begins at the time of initial HIV infection

**DOI:** 10.1016/j.isci.2022.104488

**Published:** 2022-06-30

**Authors:** Elizabeth Crabb Breen, Mary E. Sehl, Roger Shih, Peter Langfelder, Ruibin Wang, Steve Horvath, Jay H. Bream, Priya Duggal, Jeremy Martinson, Steven M. Wolinsky, Otoniel Martínez-Maza, Christina M. Ramirez, Beth D. Jamieson

**Affiliations:** 1 Cousins Center for Psychoneuroimmunology, Department of Psychiatry and Biobehavioral Sciences, David Geffen School of Medicine at UCLA, University of California Los Angeles, Los Angeles, CA 90095, USA; 2 Division of Hematology-Oncology, Department of Medicine, David Geffen School of Medicine at UCLA, University of California Los Angeles, Los Angeles, CA 90095, USA; 3 Center for Neurobehavioral Genetics, Jane and Terry Semel Institute for Neuroscience and Human Behavior, University of California Los Angeles, Los Angeles, CA 90095, USA; 4 Department of Psychiatry and Biobehavioral Sciences, David Geffen School of Medicine at UCLA, University of California Los Angeles, Los Angeles, CA 90095, USA; 5 Department of Epidemiology, Johns Hopkins Bloomberg School of Public Health, Baltimore, MA 21205, USA; 6 Department of Human Genetics, David Geffen School of Medicine at UCLA, University of California Los Angeles, Los Angeles, CA 90095, USA; 7 Altos Labs, San Diego, CA 92121, USA; 8 Department of Molecular Microbiology and Immunology, Johns Hopkins Bloomberg School of Public Health, Graduate Program in Immunology, Johns Hopkins School of Medicine, Baltimore, MD 21205, USA; 9 Department of Infectious Diseases and Microbiology, Graduate School of Public Health, University of Pittsburgh, Pittsburgh, PA 15261, USA; 10 Division of Infectious Diseases, Northwestern University Feinberg School of Medicine, Chicago, IL 60611, USA; 11 Departments of Obstetrics & Gynecology and Microbiology, Immunology, & Molecular Genetics, David Geffen School of Medicine at UCLA, University of California Los Angeles, Los Angeles, CA 90095, USA; 12 Fielding School of Public Health, University of California Los Angeles, Los Angeles, CA 90095, USA

**Keywords:** Immunology, Human physiology, Epigenetics, Virology

## Abstract

Living with HIV infection is associated with early onset of aging-related chronic conditions, sometimes described as accelerated aging. Epigenetic DNA methylation patterns can evaluate acceleration of biological age relative to chronological age. The impact of initial HIV infection on five epigenetic measures of aging was examined before and approximately 3 years after HIV infection in the same individuals (n=102). Significant epigenetic age acceleration (median 1.9–4.8 years) and estimated telomere length shortening (all p*≤* 0.001) were observed from pre-to post-HIV infection, and remained significant in three epigenetic measures after controlling for T cell changes. No acceleration was seen in age- and time interval-matched HIV-uninfected controls. Changes in genome-wide co-methylation clusters were also significantly associated with initial HIV infection (p≤ 2.0 × 10^−4^). These longitudinal observations clearly demonstrate an early and substantial impact of HIV infection on the epigenetic aging process, and suggest a role for HIV itself in the earlier onset of clinical aging.

## Highlights

• Accelerated epigenetic aging begins within three years after initial HIV infection
• No epigenetic aging was observed in age-matched men over the same time period
• T cell changes after HIV infection did not account for all epigenetic changes
• Initial HIV infection is associated with significant genomic methylation changes

## Graphical abstract

**Figure:**
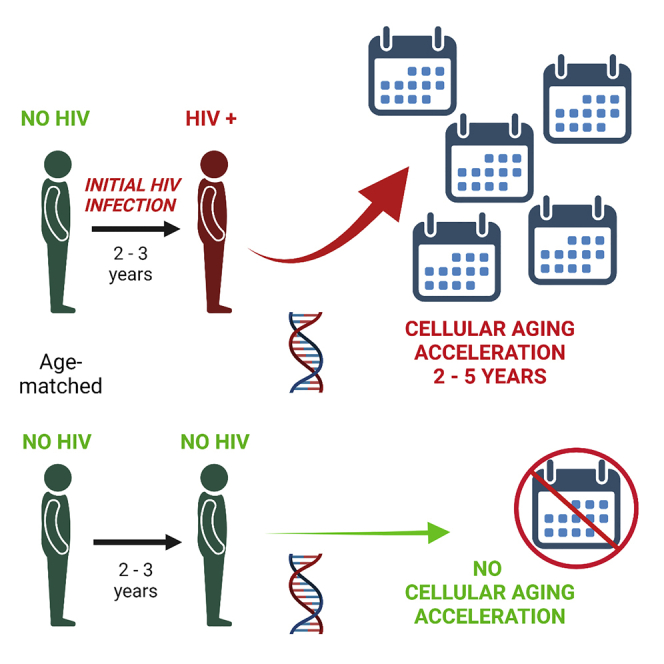

## Introduction

Despite a significant increase in life expectancy(Marcus et al., 2016; Wandeler et al., 2016; Teeraananchai et al., 2017), there is mounting evidence that living long-term with Human Immunodeficiency Virus (HIV) and antiretroviral therapy, even when clinically well-controlled, is associated with an earlier than expected onset of chronic conditions such as heart and kidney disease, frailty, and neurocognitive difficulties (Currier et al., 2003; Obel et al., 2007; Lucas et al., 2008; Desquilbet et al., 2007; Sacktor et al., 2002; Schouten et al., 2011). It has been suggested that this represents premature or accelerated aging, but consensus on this point has been hampered by a lack of agreement on, or methods by which to define, what constitutes normal aging (High et al., 2012).

In recent years, there has been tremendous interest in assessing the process of human aging at a subcellular level by examining patterns of DNA methylation (DNAm) in various cell types and tissues. This approach was pioneered by the “epigenetic clock” developed by Horvath (Horvath, 2013), which uses methylation patterns from a carefully-curated set of 353 methylation sites found in genomic DNA (known as CpGs), to predict an epigenetic or biological age that is closely correlated to chronologic age across different normal tissues in human and non-human primate species. The original Horvath clock (Pan-Tissue Clock) has been validated in a wide range of human tissues and cell types throughout the body, including peripheral blood mononuclear cells (PBMC) (Horvath, 2013). Horvath then developed acceleration measures including the intuitive “age acceleration difference,” which is calculated by subtracting the individual’s chronologic age from his or her epigenetic age, and therefore, equals the number of years that a person’s epigenetic age differs from their chronologic age. If the epigenetic age is older (a positive value using the age acceleration difference), this is viewed as an indicator of accelerated biological aging relative to chronologic age. Furthermore, an age-adjusted “age acceleration residual” (AAR), can be calculated from a linear regression model between the Horvath DNAm age and chronologic age, with greater values indicating accelerated biological aging. Another epigenetic clock was developed by Hannum (Hannum et al., 2013), and used to construct a calculated age-adjusted residual known as “extrinsic epigenetic age acceleration” (EEAA), which is positively correlated with senescent T lymphocytes and negatively correlated with naive T lymphocytes (Chen et al., 2016). In recent years, additional epigenetic measures have been developed utilizing various approaches to identify clusters of CpGs that yield “clocks” that predict differences relative to not only lifespan (years of life, “Grim epigenetic age acceleration” [GEAA]), but also healthspan (years of healthy life, “phenotypic epigenetic age acceleration” [PEAA]) (Levine et al., 2018; Lu et al., 2019a), or that estimate telomere length (TL) on chromosomes(Lu et al., 2019b), a well-documented cellular indicator of aging which becomes shorter with age. These DNA methylation-based measures (described in more detail in Table S1) provide tools by which aging at the cellular level can be evaluated in an objective manner.

In persons with HIV (PWH), some of these epigenetic approaches have been utilized to explore the possibility of accelerated biological aging in both untreated and treated HIV infection. However, epigenetic and telomere length studies thus far examining HIV infection and accelerated aging have largely been cross-sectional, comparing persons with established or chronic HIV infection to HIV-uninfected persons of the same chronologic age(Horvath and Levine, 2015; Rickabaugh et al., 2015; Gross et al., 2016; Zanet et al., 2014), rather than in longitudinal studies of the same persons over the course of initial HIV infection. These cross-sectional studies have demonstrated significant age acceleration in PWH compared to uninfected controls, utilizing a variety of approaches, including calculating the Horvath AAR, or a different calculation based on a consensus of the Horvath and Hannum epigenetic clocks, by directly measuring TL, or conducting a broad survey of genome-wide CpG sites known as Weighted Gene Correlation Network Analysis (WGCNA). In one small study of 31 intravenous drug users before and after HIV seroconversion, nearly all of whom were co-infected with Hepatitis C Virus (HCV), TL was shortened dramatically after HIV infection, but age acceleration as measured by AAR was only shown to positively correlate with time since the pre-HIV sample (Leung et al., 2017). There were no comparisons over the same time period to control subjects who were not HIV and/or HCV-infected, and no truly longitudinal analyses were reported evaluating the within-person change in AAR from pre-to post-HIV seroconversion. These studies leave open for investigation the potential contribution of lifestyle and other factors besides HIV to accelerated aging. There remains a clear need to evaluate the role of HIV infection in true longitudinal studies, especially the impact of initial acute HIV infection versus other potential variables, in accelerated biological aging utilizing an appropriate control group.

In the first ever study of this size and design, we have examined epigenetic aging over the course of initial HIV infection in more than one hundred persons, with five epigenetic measures of biological aging within six months or less before HIV infection, and again in the same persons shortly after initial HIV infection. In comparison, we evaluated the same epigenetic measures over matched time intervals in persons of the same chronologic age who did not become HIV-infected over the course of this study. The persons who became HIV-infected and those who remained uninfected were all drawn from the same population of men at-risk for HIV (men who have sex with men), and all had extensive information on other factors that might contribute to accelerated aging, regardless of HIV status. In addition to the five specific epigenetic measures, we conducted a genome-wide survey of methylation changes over the course of initial HIV infection.

To our knowledge, this is the first truly longitudinal case/control study to analyze the impact of initial HIV infection on epigenetic age, utilizing multiple DNAm-based measures and comparing the results to well-matched controls aging in the absence of HIV. In conjunction with the wealth of other clinical and demographic data available, we tested our hypothesis that initial HIV infection, and specifically the HIV viral load, would be major contributing factors to early accelerated epigenetic aging, as characterized by multiple DNAm patterns which develop in the first years of living with HIV. Likewise, we believe that this is the first longitudinal examination of genome-wide DNAm changes associated with initial HIV infection.

## Results

### Demographics

Participants from the Multicenter AIDS Cohort Study (MACS) who were included in the current substudy of initial HIV infection were predominantly white, non-Hispanic, college-educated men who have sex with men (Table 1), similar to the overall MACS demographics at the initiation of the study in the 1980s(Kaslow et al., 1987). There were slightly more non-white men among the persistently HIV-seronegative (SN) participants (26/102) compared to the participants who became HIV-infected and seroconverted (SC; 13/102, p = 0.02), which is consistent with the larger MACS biomarker study from which the participants for this substudy were drawn (Wada et al., 2015). The SN and SC groups did not differ significantly by Hispanic ethnicity or level of education (minimum p values > 0.3). Mean age in SC and SN at Visit B (post-HIV infection or equivalent visit) was 39.0 and 38.5 years, respectively, reflecting the matching criteria (range 22–72 years across all participants). Likewise, because of matching criteria, the percentage of SC and SN with HCV infection were similar and small (2–3%), and both groups had low rates of active Hepatitis B Virus (HBV) infection at both visits (1–3%). As expected, based on reports of ≥90% Cytomegalovirus (CMV) seroprevalence among homosexual men(Drew et al., 1981; Nerurkar et al., 1987), the MACS men in this substudy for whom CMV serostatus data were available showed very high CMV seropositivity (97–100%) at Visit A, when both SC and SN were HIV-uninfected. Consistent with a previous MACS report among HIV-seropositive participants(Akhtar-Khaleel et al., 2016), SC had more cumulative pack-years of smoking than SN (p = 0.03 by Visit B).

**Table 1:** Demographics and characteristics of HIV seroconverter (SC) and matched HIV seronegative (SN) participants from the Multicenter AIDS Cohort Study (MACS)

Participants	SC^a^, n (%) or mean (SD)	SN^b^, n (%) or mean (SD)
White Race	89 (87.3%)	76 (74.5%)
Non-Hispanic Ethnicity	95 (93.1%)	92 (90.2%)
>1 year of College Education	91 (89.2%)	86 (84.3%)
Visit A to Visit B, years	2.9 (0.5)	2.7 (0.7)

At each visit	Visit A	Visit B	Visit A	Visit B
Age, years	35.7 (8.2)	38.5 (8.0)	36.3 (8.4)	39.0 (8.1)
Hepatitis C Virus RNA-positive	2 (2.0%)	3 (2.9%)	2 (2.0%)	3 (2.9%)
Hepatitis B Virus surface antigen-positive	2 (2.0%)	1 (1.0%)	2 (2.0%), n=99	3 (2.9%), n=100
Cytomegalovirus antibody-positive	90 (100%), n=90	N/A	64 (97%), n=66	N/A
Body Mass Index, kg/m^2^	24.1 (3.1)	24.7 (3.3), n=97	25.2 (5.7), n=100	25.8 (4.2), n=97
Smoking, cumulative pack years	12.0 (15.4), n=101	13.1 (16.5), n=98	7.8 (13.3)	8.3 (13.8), n=101
Absolute CD4 T cell count, cells/mm^3^	1087 (384), n=93	616 (241), n=100	1004 (428), n=97	1000 (365), n=93
Plasma HIV Viral Load^c^, copies/mL	N/A	49,757 (121,315)	N/A	N/A
Estimated time since HIV infection^d^, years	N/A	2.2 (0.5)	N/A	N/A

a n=102 SC at Visit A and Visit B unless indicated otherwise

b n=101 SN at Visit A, n=102 at Visit B unless indicated otherwise

c For 10 SC missing HIV Viral Load (VL) at Visit B, HIV VL from the closest MACS study visit 3-6 months prior to Visit B was used

d Date of HIV infection estimated as midpoint between last MACS study visit that was HIV seronegative and HIV VL undetectable (if VL data were available) and the first MACS study visit with either HIV-positive serostatus or detectable HIV VL, whichever came first

By design, the mean time intervals between peripheral blood mononuclear cell (PBMC) samples at Visits A and B were very similar in SC (2.9 years, range 1.3–3.7), and SN (2.7 years, range 0.9–4.0). Mean absolute CD4 T cell counts were stable in SN from Visit A to B at approximately 1000 cells/mm^3^, and similar to the mean CD4 counts seen in SC at the pre-HIV infection visit (Visit A, 1087 cells/mm^3^, p = 0.2). As expected, after initial HIV infection, the SC showed dramatically lower mean CD4 T cells at Visit B (mean = 616 cells/mm^3^, p < 0.001), but also had a wide range (73–1210 cells/mm^3^).

Among SC, the mean time interval between estimated date of HIV infection and the post-HIV infection PBMC sample at Visit B was 2.2 years (range 0.7–3.3 years). The calendar dates of estimated HIV infection ranged from 1985–2006, with 86% of the infection dates before 1995; regardless of calendar time, all SC post-HIV infection samples were before the initiation of highly active antiretroviral therapy (HAART) (Castillo-Mancilla et al., 2016). Mean plasma HIV viral load (VL) in SC at or immediately preceding Visit B was 49,757 copies/mL (median 14,084 copies/mL), ranging from a single individual with <50 copies/mL up to 948,000 copies/mL.

### Multiple epigenetic age acceleration measures differ significantly after initial HIV infection

**Figure 1:**
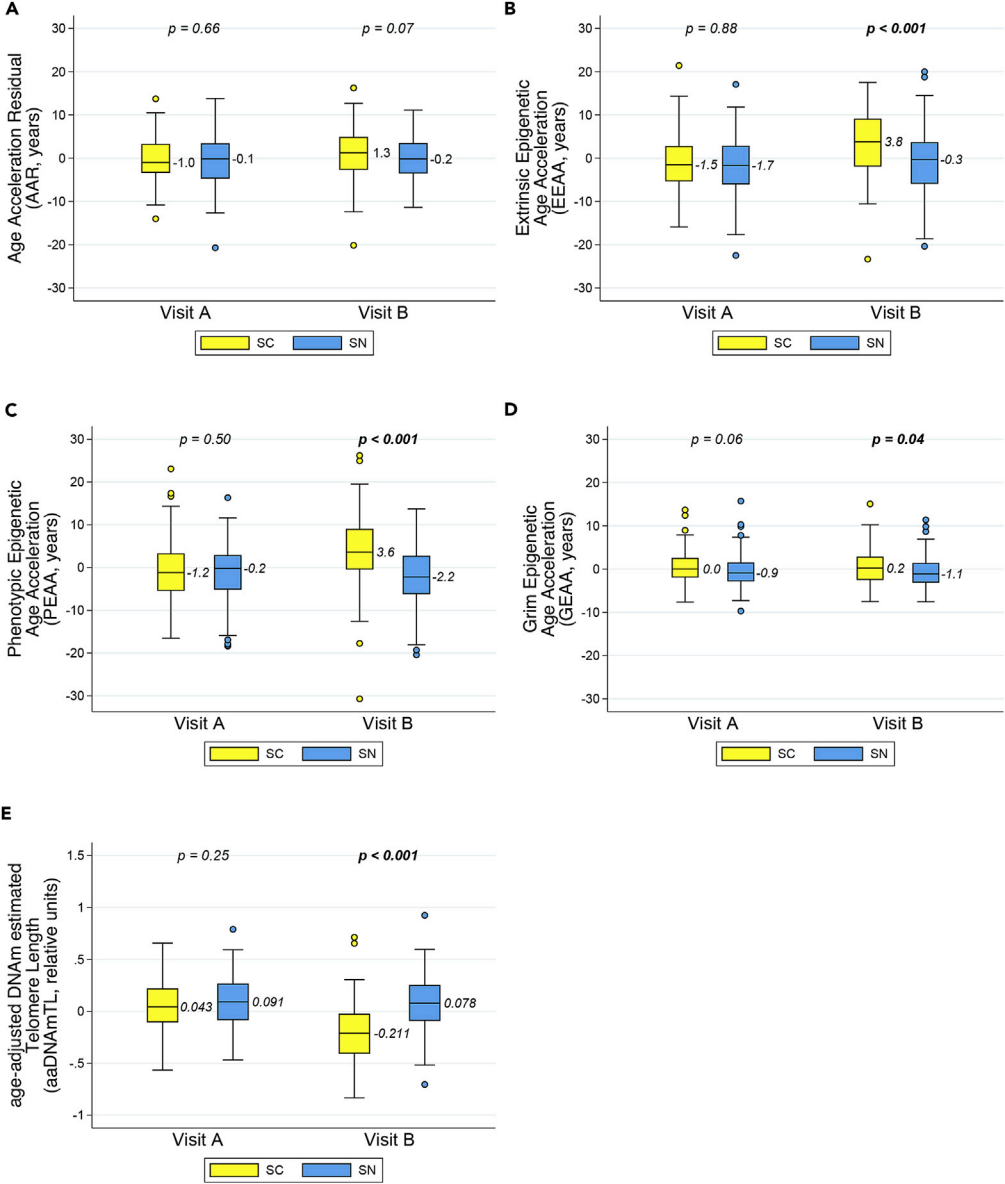
Multiple epigenetic measures in peripheral blood mononuclear cells (PBMC) demonstrate significant differences in biological aging after initial HIV infection, compared to age-matched HIV-uninfected persons Longitudinal PBMC samples from men before (Visit A) and after (Visit B) documented HIV infection and seroconversion (SC), and from matched (chronologic age, Hepatitis C status, and time interval) persistently HIV seronegative men (SN), were evaluated for biological aging by five different age-adjusted epigenetic measures: (A) Age Acceleration Residual (AAR), (B) Extrinsic Epigenetic Age Acceleration (EEAA), (C) Phenotypic Epigenetic Age Acceleration (PEAA), (D) Grim Epigenetic Age Acceleration (GEAA), and (E) age-adjusted DNA methylation-based estimate of telomere length (aaDNAmTL) (see also Table S1). The first four are epigenetic “clocks” which increase with aging whereas estimated TL shortens (decreases) with aging. Each panel shows box and whisker plots (heavy line = median, box = 25^th^-75^th^ percentile, whiskers = 5^th^-95^th^ percentile) for SC (yellow) and SN (blue) participants at Visit A and Visit B; p values are for comparison of SN vs. SC at each visit by t-tests. 102 matched SC/SN pairs were evaluated; one SN participant was missing a PBMC sample at Visit A.

At Visit A, when all participants were HIV-uninfected, both the SC and SN groups had median epigenetic ages that differed from chronologic age by 1 year or less, as calculated by the Age Acceleration Residual (AAR, Figure 1A). When comparing the SC (before they became HIV-infected) and matched SN (those who remain HIV-uninfected) to each other at Visit A, there were no statistically significant differences between the two groups in the AAR, Extrinsic Epigenetic Age Acceleration (EEAA), and Phenotypic Epigenetic Age Acceleration (PEAA) clocks (all p > 0.50, Figures 1A–1C). The Grim Epigenetic Age Acceleration (GEAA) clock demonstrated a small difference bordering on statistical significance (p = 0.06; Figure 1D), with the SC group being slightly epigenetically older than the SN group. Age-adjusted DNA methylation-based estimates of telomere length (aaDNAmTL) were similar in the SC and SN at Visit A (p = 0.25, Figure 1E).

At Visit B, following initial HIV infection in the SC, SC showed dramatically significant differences in epigenetic age acceleration (greater EEAA, PEAA) and estimated telomere length (shorter aaDNAmTL) compared to matched HIV-uninfected SN (all p values < 0.001), and continued to be slightly more accelerated in GEAA (p = 0.04) (Figures 1B–1E). Median AAR was greater in SC compared to SN, but the differences were marginally non-significant (p = 0.07, Figure 1A).

**Figure 2:**
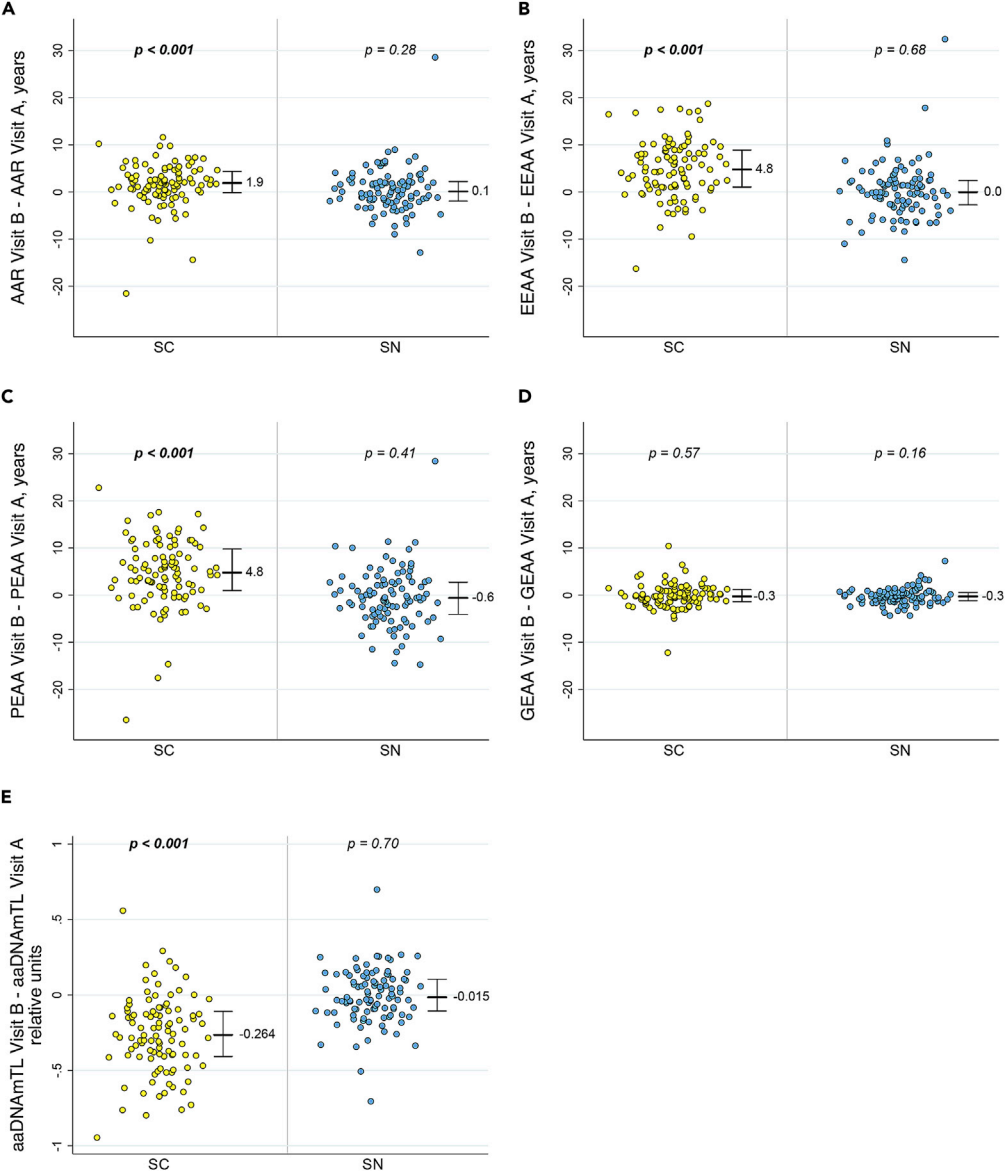
Significant accelerations in multiple epigenetic measures of aging occur in men over the course of initial HIV infection, but not in matched men who remain HIV-uninfected Dot plots of HIV seroconverter (SC, n = 102) and persistently HIV seronegative (SN, n = 101) participants show the epigenetic change from the pre-HIV infection or equivalent visit (Visit A) to the post-HIV infection or equivalent visit (Visit B) within each participant as measured by (A) AAR, B) EEAA, (C) PEAA, (D) GEAA, and (E) aaDNAmTL. Heavy bar and numerical value = median change, whiskers = 25^th^-75^th^ percentiles, p values = t-test for change within each participant group for differences from zero.

When epigenetic changes were evaluated within each persistently HIV-uninfected (SN) individual over the time interval between Visits A and B (just under 3 years on average), no significant changes were seen in any of the epigenetic clocks (−0.6 to 0.1 years median change, p ≥ 0.16) or in the aaDNAmTL (median difference −0.015 relative units, p = 0.70) (Figures 2A–2E). This lack of demonstrable age acceleration over approximately three years in multiple measures of epigenetic aging is consistent with previous reports based on the original Horvath clock, indicating the resolution of that epigenetic clock is between 3 and 4 years(Horvath, 2013).

In sharp contrast, over matched time intervals between Visits A and B among SC experiencing initial HIV infection, AAR, EEAA, and PEAA clocks showed 1.9, 4.8, and 4.8 median years age acceleration at Visit B, respectively (all p*≤* 0.001, Figures 2A–2C). Likewise, aaDNAmTL showed significant shortening, with a median estimated change of −0.264 relative units from Visit A to Visit B (p*<* 0.001, Figure 2E), which is 17.6-fold greater than the median estimated change seen in SN. GEAA in SC showed very little change (−0.3 year median difference, p = 0.57) which was the same as in the SN (−0.3 year, p = 0.16, Figure 2D).

### Initial HIV infection remains associated with older EEAA and PEAA ages, and shorter estimated aaDNAmTL, even after taking demographic factors and T cell changes into account

Mixed model analyses on all participants at both visits, taking demographic characteristics into account, demonstrated that becoming infected with HIV between Visits A and B contributed to epigenetic aging as measured by EEAA, PEAA and aaDNAmTL (all p < 0.001), with no significant contribution to these three measures made by HBV status, body mass index (BMI), or smoking (all p≥ 0.18, Table 2). Study visit (A or B) alone strongly contributed to the chronologic age-adjusted epigenetic values over time for AAR, EEAA, PEAA, and aaDNAmTL (all p ≤ 0.001) but not GEAA (Table 2). However, when examining the interaction of the study visit and HIV serostatus group (i.e., SC or SN), which describes the change in HIV infection status from HIV-uninfected to HIV-infected in the SC group compared to the persistently HIV-uninfected SN group, initial HIV infection was clearly the driving force behind the epigenetic values seen at the two visits for EEAA, PEAA, and aaDNAmTL. AAR, even though it changed significantly as a function of study visit, had no associations with any of the other co-variates, including HIV serostatus group or the study visit∗HIV serostatus group interaction (all p ≥ 0.16). Race was not a significant contributor for AAR, EEAA, or PEAA, but was strongly significant for GEAA (p ≤ 0.001) and weakly significant for aaDNAmTL (p = 0.03). Further exploration of GEAA revealed that this effect was driven by the small subpopulation of non-white participants (n = 39 non-whites out of 203 total participants), especially in the SC group (13 non-whites out of 102 SC, 3.1 years age acceleration because of non-white race alone, p = 0.01, data not shown). For aaDNAmTL, white and non-white participants differed slightly in mean estimated aaDNAmTL at Visit A (p = 0.03), but they did not differ significantly at Visit B (p = 0.10, data not shown). However, the aaDNAmTL values among the 39 non-white participants at Visit A were not normally distributed. Although it is of great interest, because of the limitations of statistical power as a result of small numbers of non-white individuals, it was not feasible to explore any additional relationships between race and either GEAA or aaDNAmTL. As expected, smoking history was strongly associated with GEAA, because of the incorporation of smoking-related biomarkers into this particular epigenetic clock (Table S1). Results from the mixed model fixed effects analyses for individual co-variates for all five epigenetic measures are shown in Table S2, and were consistent with models taking all co-variates into account.

**Table 2:** Potential contribution of demographic and behavioral co-variates to epigenetic measures over time, using mixed effects models

Potential Contributors to Epigenetic Measures	F value (p *value*)^a^				
	AAR	EEAA	PEAA	GEAA	aaDNAmTL
Study Visit, Visit A vs B	11.00 (*0*.*001*)	38.85 (*<0*.*001*)	21.01 (*<0*.*001*)	1.56 *(0*.*21)*	75.13 (*<0*.*001*)
HIV Serostatus Group, SC vs SN^b^	1.99 (*0*.*16*)	5.29 (*0*.*02*)	10.22 (*0*.*002*)	3.39 *(0*.*07)*	15.14 (*<0*.*001*)
Study Visit∗HIV Serostatus Group	1.66 *(0*.*20)*	29.68 *(<0*.*001)*	26.23 *(<0*.*001)*	0.00 *(0*.*96)*	63.44 *(<0*.*001)*
Race, non-white vs white	0.36 (*0*.*55*)	1.70 (*0*.*19*)	3.37 (*0*.*07*)	30.38 *(<0*.*001)*	4.52 (*0*.*03*)
Hepatitis B Status, HBsAg – vs + c	1.22 (*0*.*27*)	0.19 (*0*.*66*)	0.14 (*0*.*71*)	0.10 *(0*.*75)*	0.70 (*0*.*40*)
BMI, kg/m^2^	0.02 (*0*.*89*)	0.17 (*0*.*68*)	0.10 (*0*.*75*)	0.79 *(0*.*38)*	0.15 (*0*.*70*)
Smoking, cumulative pack years	0.01 (*0*.*93*	0.03 (*0*.*87*)	1.79 (*0*.*18*)	47.15 *(<0*.*001)*	1.5 (*0*.*22*)

AAR = Age-Acceleration Residual, EEAA = Extrinsic Epigenetic Age Acceleration, PEAA = Phenotypic Epigenetic Age Acceleration, aaDNAmTL = age-adjusted DNA methylation-based estimate of Telomere Length, HBsAg = Hepatitis B surface Antigen, BMI = Body Mass Index.

a F values and Pr > F p values (p values in italics, bold if < 0.05) from mixed models incorporating all potential co-variates for all participants at both visits (n = 387 out of 407 total observations due to missing data for some co-variates) in a single model (see also Table S2).

b HIV serostatus groups classified as SC (became HIV-infected and seroconverted between Visits A and B) vs SN (persistently HIV-uninfected and seronegative at Visits A and B).

c Hepatitis B virus status classified by current HBsAg at visit, negative vs positive.

Separately from the mixed models, pairwise correlations among the four epigenetic clocks and the estimated aaDNAmTL were evaluated (Table S3). When including all participants at Visit A (all HIV-uninfected, n = 203), every pairwise correlation among all the measures was highly significant (p ≤ 0.003). This was not surprising because all were intended to examine epigenetic or biological aging, even though they were developed separately, each using a different curated set of CpG methylation sites. When evaluating the correlations in SC and SN groups separately at Visit B (n = 102 each group), all measures remained significantly correlated except GEAA, which was no longer correlated with AAR in the HIV-infected SC, and was correlated only with PEAA in the uninfected SN.

As noted in Table 1, the SC group showed a large decrease in mean absolute CD4 T cell numbers at Visit B, as expected following HIV infection. Therefore, it was appropriate to develop another set of mixed models that could account for potential differences in the cell composition of each sample. Multiple methods have been developed for utilizing DNAm data to estimate the proportion of different cell types in blood samples, including those of Houseman(Houseman et al., 2012), and of Salas(Salas et al., 2018), which draws at least in part on Houseman’s method. To be consistent with the approach that was utilized to calculate values for the epigenetic measures on study samples, the same software platform http://dnamage.genetics.ucla.eduwas initially utilized to impute cell proportions for each sample, which is based on Houseman’s method(Horvath et al., 2016; Horvath and Levine, 2015). However, these imputed proportions might suffer from the underestimation of CD4 and/or overestimation of CD8 T cells that was recently described among HIV-infected subjects when using the Houseman method(Shu et al., 2020). As an alternative, direct measures of absolute numbers of CD4 and CD8 T cells were available from the MWCCS database, and measurements of selected T cell subsets by flow cytometry were performed at UCLA on a portion of each of the thawed viable PBMC samples from which DNA had been extracted for DNAm analyses. Therefore, the absolute numbers of total CD4 and CD8 T cells, and of naïve, activated, and senescent CD4 and CD8 T cell subsets, which are likely to be more reliable than imputed proportions, were available for use in analyses for most participants at both visits (Table 3). As expected, there were no differences in any of the mean absolute T cell counts between the SC and SN group at Visit A (all HIV-uninfected), but significant differences at Visit B in 6 out of the 8 T cell subsets (all but activated and senescent CD4 T cells). Similarly, mean within-person changes in absolute T cell numbers from Visit A to Visit B showed no changes in SN, and highly significant changes in the same 6 T cell subsets in SC (Table S4). Correlations between absolute counts of different T cell subsets, and between each T cell subset and the five epigenetic measures, are shown in Tables S5–S7.

**Table 3:** Mean absolute T cell counts of the SC and SN groups, at Visits A and B

T cell population^a^	Visit A^b^, Mean (SE) n			Visit B^c^, Mean (SE) n		
	SC	SN	*p value*^d^	SC	SN	*p value*^d^
CD4 T cells, cells/mm^3^	1088 (384) n = 93	1004 (428) n = 97	*0*.*16*	616 (240) n = 101	1000 (365) n = 93	*< 0*.*001*
CD8 T cells, cells/mm^3^	630 (252) n = 93	598 (287) n = 97	*0*.*42*	890 (402) n = 101	617 (295) n = 93	*< 0*.*001*
Naive (CD45RA^+^CCR7^+^) CD4 T cells, cells/mm^3^	404 (193) n = 92	406 (293) n = 96	*0*.*95*	244 (140) n = 101	383 (231) n = 92	*< 0*.*001*
Naive (CD45RA^+^CCR7^+^) CD8 T cells, cells/mm^3^	219 (116) n = 92	212 (130) n = 96	*0*.*72*	138 (82) n = 101	214 (113) n = 92	*< 0*.*001*
Activated (HLA-DR^+^CD38^+^) CD4 T cells, cells/mm^3^	28 (13) n = 90	27 (19) n = 95	*0*.*63*	32 (17) n = 99	28 (18) n = 90	*0*.*14*
Activated (HLA-DR^+^CD38^+^) CD8 T cells, cells/mm^3^	25 (16) n = 90	23 (20) n = 95	*0*.*40*	180 (149) n = 99	26 (30) n = 90	*< 0*.*001*
Senescent (CD28^−^CD57^+^) CD4 T cells, cells/mm^3^	45 (55) n = 92	33 (42) n = 96	*0*.*10*	39 (50) n = 101	33 (37) n = 92	*0*.*34*
Senescent (CD28^−^CD57^+^) CD8 T cells, cells/mm^3^	108 (81) n = 92	104 (88) n = 96	*0*.*79*	140 (115) n = 101	105 (105) n = 92	*0*.*03*

a Absolute CD4 and CD8 T cell counts obtained from MWCCS database, and were determined by standardized flow cytometry at the time of original blood sample collection; T cell subsets determined by multicolor flow cytometry at the time of thawing of viable PBMC aliquots as described in the STAR Methods, and absolute T cell subset counts calculated from total CD4 and CD8 counts (see also Table S4).

b All participants HIV-uninfected at Visit A, matched on age and hepatitis C status.

c SC recently HIV-infected, SN persistently HIV-uninfected at matched time intervals at Visit B.

d p values are for comparison of SC vs. SN at each visit by t-tests (p values in italics, bold if < 0.05).

Utilizing different combinations of T cell subsets, as described in the STAR Methods, a consensus model was identified with the best fit across all five of the epigenetic measures. This model included natural log-transformed absolute T cell counts for total CD4, total CD8, naïve CD4, activated CD8, and senescent CD8, and was utilized to analyze the possible contributions of changes over time in cell numbers, as well as changes in HIV infection status, to the observed values for each epigenetic measure (Table 4, Table S8). Significant associations were observed between T cell subset counts and one or more of the epigenetic measures, and all measures but AAR were significantly associated independently with study visit and with HIV serostatus group (Table 4). However, even when controlling for five T cell subsets, the same three epigenetic measures that were significant in the original mixed model, EEAA, PEAA, and estimated aaDNAmTL, still showed a statistically significant relationship to the interaction of study visit∗HIV serostatus group (mixed effects models, p = 0.03, 0.04, <0.001, respectively), thus confirming initial HIV infection that occurs between Visit A and Visit B, but only in the SC group, as contributing to epigenetic age acceleration by these measures. As further confirmation, the frequencies of T cell subsets within the lymphocyte population were calculated using the flow cytometry measurements directly made on the PBMC samples, at Visits A and B for SC and SN groups (Table S9), and the consensus mixed model was repeated for each of the five epigenetic measures (Table S10). Similar to the results with absolute cell counts, significant associations were observed between T cell subset percentages and one or more of the epigenetic measures, and independently with study visit and HIV serostatus group. Most importantly, consistent with the original mixed models (Table 2) and the models taking absolute cell numbers into account (Table 4), EEAA, PEAA, and estimated aaDNAmTL remained significantly associated with the study visit∗HIV serostatus group interaction that describes initial HIV infection, even after controlling for changes in percentages of T cell subsets (p = 0.04, 0.01, <0.001, respectively, Table S10).

**Table 4:** Potential contribution of absolute T cell counts to epigenetic measures over time, using mixed effects models

Potential Contributors to Epigenetic Measures	F value (p *value*)^a^				
	AAR	EEAA	PEAA	GEAA	aaDNAmTL
Study Visit, Visit A vs B	0.49 *(0*.*48)*	9.12 *(0*.*003)*	4.86 *(0*.*03)*	5.18 *(0*.*02)*	11.98 *(<0*.*001)*
HIV Serostatus Group, SC vs SN^b^	3.52 *(0*.*06)*	9.77 *(0*.*002)*	16.07 *(<0*.*001)*	5.62 *(0*.*02)*	37.97 *(<0*.*001)*
Study Visit∗HIV Serostatus Group	0.14 *(0*.*71)*	4.90 *(0*.*03)*	4.09 *(0*.*04)*	0.68 *(0*.*41)*	15.40 *(<0*.*001)*
CD4 T cells^c^, ln cells/mm^3^	2.51 *(0*.*11)*	7.18 *(0*.*008)*	5.56 *(0*.*02)*	4.85 *(0*.*03)*	15.28 *(<0*.*001)*
CD8 T cells, ln cells/mm^3^	0.02 *(0*.*89)*	4.59 *(0*.*03)*	1.24 *(0*.*27)*	0.98 *(0*.*32)*	5.63 *(0*.*02)*
Naive (CD45RA^+^CCR7^+^) CD4 T cells, ln cells/mm^3^	7.41 *(0*.*007)*	21.3 *(<0*.*001)*	14.89 *(<0*.*001)*	1.06 *(0*.*30)*	18.63 *(<0*.*001)*
Activated (HLA-DR^+^CD38^+^) CD8 T cells, ln cells/mm^3^	6.59 *(0*.*01)*	1.94 *(0*.*17)*	2.68 *(0*.*10)*	0.02 *(0*.*90)*	1.80 *(0*.*18)*
Senescent (CD28^−^CD57^+^) CD8 T cells, ln cells/mm^3^	16.71 *(<0*.*001)*	15.95 *(<0*.*001)*	5.50 *(0*.*02)*	0.95 *(0*.*33)*	25.61 *(<0*.*001)*

AAR = Age-Acceleration Residual, EEAA = Extrinsic Epigenetic Age Acceleration, PEAA = Phenotypic Epigenetic Age Acceleration, aaDNAmTL = age-adjusted DNA methylation-based estimate of telomere length.

a F values and Pr > F p values (p values in italics, bold if < 0.05) from mixed models incorporating all potential co-variates for all participants at both visits (n = 374 out of 407 total observations due to missing data for some co-variates) in a single model (see also Table S8).

b HIV serostatus groups classified as SC (became HIV-infected and seroconverted between Visits A and B) vs SN (persistently HIV-uninfected and seronegative at Visits A and B).

c Absolute counts of T cell subsets as described in STAR Methods and Table S13; all cell counts natural log-transformed (ln) for analyses.

### Plasma HIV viral load correlates with EEAA and PEAA clocks, and estimated aaDNAmTL at the post-HIV infection visit

The persistent significant associations between three of the epigenetic measures and the study visit∗HIV serostatus group interaction, representing the HIV infection event in the SC group, even after accounting for demographic factors or T cell changes, suggests a role for HIV itself in contributing to accelerated biological aging. Regression analyses were performed to determine if the amount of circulating HIV present post-infection, i.e., the plasma HIV viral load (VL), was correlated with the magnitude of any of the epigenetic measures at Visit B. Consistent with the mixed models, greater epigenetic age acceleration, as measured by higher EEAA and PEAA (both p = 0.002), and shorter estimated telomere length, as measured by aaDNAmTL (p = 0.025), were significantly associated with higher HIV VL post-HIV infection whereas neither AAR nor GEAA were correlated with HIV VL (Figures S1A–S1E). For every log_10_ increase in HIV VL, EEAA and PEEA increased 2.6 and 3.1 years, respectively. For every log_10_ increase in HIV VL, aaDNAmTL shortened by 0.075 relative units (28% of the median change in SC from Visit A to B). As expected, following initial HIV infection, HIV VL and absolute CD4 T cell counts were inversely correlated at Visit B (correlation coefficient = −0.26; p = 0.01). Because HIV VL and absolute CD4 counts were not independent and exhibited high correlation, when both HIV VL and CD4 were included in the analyses, neither co-variate showed a significant association with any of the epigenetic measures, which can occur with multicollinearity (all p values > 0.05, data not shown).

### Genome-wide methylation of CpGs changes significantly with initial HIV infection

Weighted Gene Correlation Network Analysis (WGCNA) (Langfelder and Horvath, 2008) was utilized to identify clusters of CpGs that are correlated with each other across all of the samples analyzed (co-methylation modules), using methylation levels measured at over 850,000 individual CpG methylation sites on the Infinium MethylationEPIC BeadChip. Sixty-seven co-methylation modules were identified, 18 of which showed statistically significant mean module eigenvector methylation differences from visit A to visit B in the SC group (i.e., over the course of initial HIV infection, p values ≤ 2.0 × 10^−4^, Table 5), but none of the 67 modules showed significant differences between visits in the SN group (data not shown). For each CpG within each of the 18 HIV infection-associated modules, we calculated an intramodular connectivity measure (kME value) (Horvath and Dong, 2008) and chose the most stringent cutoff of 0.85 to identify genes within each module. The number of CpG sites with kME value ≥ 0.85 ranged from 1–36,000+ (Table 5). All individual CpG sites within these 18 modules are listed in Table S11 (Excel file). Enrichment analyses were performed using the EnrichR gene list enhancement tool(Kuleshov et al., 2016) to identify overrepresented biological pathways for those CpG sites with kME ≥0.85 within the 18 modules (Table S12, Excel file). These analyses highlighted large numbers of pathways, especially in modules 2 and 18, with many related to embryonic morphogenesis and cellular differentiation, or immune function and biosynthesis, respectively.

**Table 5:** Weighted Gene Correlation Network Analysis (WGCNA) of genome-wide methylation of CpG sites that change significantly with initial HIV infection in the SC group

Co-methylation Module^a^	# of CpGs in Module	Module eigenvector methylation^b^			# (%) of CpGs with kME≥0.85^d^
		Visit A Mean (SD)	Visit B Mean (SD)	*p value*^c^	
1	133,037	0.004 (0.017)	−0.006 (0.020)	*3*.*6x10*^*−4*^	37,284 (28)
2	37,328	−0.015 (0.014)	−0.003 (0.017)	*5*.*5x10*^*−8*^	8097 (21)
3	16,985	−0.008 (0.014)	0.003 (0.022)	*5*.*5x10*^*−8*^	2300 (14)
4	9,775	0.012 (0.018)	−0.005 (0.021)	*5*.*8x10*^*−9*^	1596 (16)
5	5,615	−0.022 (0.014)	0.004 (0.017)	*3*.*2x10*^*−21*^	672 (12)
6	5,184	0.009 (0.017)	−0.002 (0.024)	*3*.*2x10*^*−4*^	892 (17)
7	2,398	−0.006 (0.023)	0.005 (0.022)	*2*.*2x10*^*−4*^	514 (21)
8	1,677	0.016 (0.016)	−0.004 (0.019)	*1*.*5x10*^*−13*^	141 (8)
9	1,019	0.006 (0.019)	−0.005 (0.021)	*3*.*7x10*^*−5*^	50 (5)
10	962	0.016 (0.016)	−0.005 (0.020)	*2*.*5x10*^*−12*^	58 (6)
11	707	−0.013 (0.023)	−0.002 (0.022)	*6*.*0x10*^*−4*^	22 (3)
12	222	−0.012 (0.025)	0.003 (0.015)	*1*.*8x10*^*−6*^	1 (0.4)
13	215	−0.017 (0.027)	−0.005 (0.025)	*2*.*0x10*^*−4*^	26 (12)
14	106	−0.009 (0.014)	0.010 (0.022)	*1*.*7x10*^*−10*^	5 (4.7)
15	94	−0.005 (0.018)	0.015 (0.023)	*1*.*1x10*^*−10*^	1 (1)
16	67	−0.015 (0.018)	−0.003 (0.020)	*2*.*8x10*^*−6*^	1 (1.5)
17	44	−0.022 (0.021)	−0.002 (0.019)	*5*.*9x10*^*−11*^	2 (5)
18	38	0.011 (0.007)	−0.003 (0.018)	*2*.*5x10*^*−11*^	17 (45)

a Each Co-methylation Module is a cluster of CpG methylation sites within the 850,00 + sites evaluated on the Infinium MethylationEPIC BeadChip, identified by WGCNA to be correlated with each other; any one CpG site belongs to only one Module. Out of a total of 67 Modules identified by WGCNA utilizing all samples from all participants at both visits (n = 407 samples), 18 Modules shown are those that are significantly associated with the change in HIV status from visit A to visit B in the SC group (initial HIV infection). Modules 1–18 are numbered according to the number of CpGs (largest to smallest) contained in each Module. All CpGs in Modules 1–18 are listed in Table S11 (Excel file).

b Methylation levels are quantified by the beta value from the EPIC BeadChip assay, using the ratio of intensities between methylated and un-methylated alleles as described in the STAR Methods. In WGCNA, a representative methylation profile for each Module, known as the Module eigenvector, is defined as the first principal component in the Module methylation matrix. Mean and SD eigenvector methylation values shown for each Module are based on 102 HIV seroconverters (SC group) with observations at both Visits A and B.

c Italicized p values are from a non-parametric group comparison test (Kruskal-Wallis) comparing mean Module eigenvector methylation from Visit A (before HIV infection) to Visit B (after initial HIV infection); level of significance for Module association with HIV infection accounting for multiple comparisons is p*< 0*.*05/67* or*<7*.*5x10*^*−4*^.

d kME is the intramodular connectivity measure for each CpG calculated from the WGCNA, and ≥0.85 is the threshold for a CpG to be considered a “hub” site, as described in the STAR Methods. All CpGs from Modules 1–18 with kME ≥0.85 were included in a pathways enrichment analysis (please see Table S12, Excel file), and Modules in bold (n = 5) contain at least one CpG in a gene that falls within biological pathways with significant p values after adjustment for multiple comparisons.

## Discussion

Although small longitudinal studies have examined accelerated aging in HIV seroconverters(Leung et al., 2017), and in perinatally HIV-infected youth many years after HIV infection(Shiau et al., 2021), and cross-sectional studies suggest that persons with HIV may be aging at a faster rate(Rickabaugh et al., 2015; Horvath and Levine, 2015; Gross et al., 2016), the study reported here is the largest, and the first with matched HIV-uninfected controls, to longitudinally follow individuals over the course of becoming infected with HIV, and to document epigenetic changes consistent with accelerated biological aging. We have utilized four well-validated epigenetic measures based on methylation patterns of genomic DNA, each of which calculates years of biological age acceleration relative to chronologic age (known as epigenetic “clocks”), and an age-adjusted DNA methylation-based estimate of the length of telomeres at the ends of chromosomes (which are known to shorten with repeated cell division and increasing age). Over a relatively short time frame (less than three years on average), during which age-matched HIV-uninfected men showed no significant changes or acceleration in any epigenetic measure of aging, men who became infected with HIV showed highly significant age acceleration in three out of four of the epigenetic clocks as well as accelerated estimated telomere shortening (Figure 2). In mixed models, taking demographic and clinical co-variates or absolute cell counts or percentages of five T cell subsets into account (Tables 2 and 4 and S10), age acceleration in two epigenetic clocks, EEAA and PEAA, and shortening in the DNAm-based estimate of TL remained significantly associated with initial HIV infection. This clearly demonstrates an early and substantial impact of HIV infection on the epigenetic aging process that begins in the first months and years of living with HIV. This result in multiple epigenetic measures is not simply due to the correlations observed between the measures, as each was developed separately. Rather, because all were constructed for the purpose of examining epigenetic or biological aging, it is not surprising that they are correlated to each other and that more than one of the epigenetic measures point to a role of initial HIV infection in age acceleration.

EEAA and PEAA consistently showed significant results associated with HIV infection, with a median biological age acceleration of 4.8 years in SC, after adjusting for chronologic age. EEAA was recently developed from the Hannum clock of 71 CpGs(Hannum et al., 2013) focusing on mortality, but was also specifically designed to be positively correlated with senescent (aged) T cells and negatively correlated with naïve T cells of the immune system (Chen et al., 2016). PEAA is also a recently-developed epigenetic clock, utilizing a phenotypic measure of mortality from 513 CpGs of Levine (Levine et al., 2018), and so predicts lifespan. In populations not infected with HIV, a one-year increase in epigenetic age acceleration as measured by EEAA and PEAA is associated with an increase in all-cause mortality risk of 4.0% and 4.5%, respectively(Chen et al., 2016; Levine et al., 2018), which would translate to approximately 20% or more increase in mortality risk with a five-year increase in epigenetic age acceleration. The 4.8 years of acceleration revealed by these two clocks shortly following initial HIV infection clearly indicates that becoming infected and living with HIV for only three years or less is already associated with approximately 20% increased risk for a shortened lifespan. AAR, which is an age-adjusted residual of the difference in years between biological and chronological age calculated from 353 CpGs by Horvath’s original Pan-Tissue epigenetic clock (Horvath, 2013), showed a more modest median acceleration of biological aging of 1.9 years over the course of initial HIV infection, but was no longer significantly-associated with HIV infection when controlling for demographic factors or T cell subsets. This further illustrates that although AAR, EEAA, and PEAA values among the PBMC samples were strongly correlated, clocks incorporating information on naive and senescent cell composition such as EEAA, and “second-generation” clocks such as PEAA focused on mortality may be detecting differential effects of the impact of initial HIV infection.

The age-adjusted DNAm-based estimate of telomere length (aaDNAmTL) (Lu et al., 2019b), characterizes accelerated biological aging in the opposite direction of the epigenetic clocks, as telomeres become shorter with repeated cellular divisions, yielding a lower value and a negative direction of change over time. Similar to acceleration in the EEAA and PEAA epigenetic clocks, aaDNAmTL showed accelerated telomere shortening over the course of initial HIV infection in SC participants, but essentially no change in SN participants over the approximately three years evaluated in this study. Because age acceleration in the EEAA clock correlates with increases in the number of senescent T cells, which are terminally-differentiated aged cells no longer able to divide, and TL is similarly linked to cell division, the strong association of both of these measures of accelerated biological aging with initial HIV infection may be due, at least in part, to rapid changes in the cells of the immune system itself.

It is of interest to note that the GEAA epigenetic clock, which was developed based on 1030 CpGs of Lu (Lu et al., 2019a) and is also focused on mortality and lifespan, showed no differences over time regardless of changes in HIV infection status. This was the only epigenetic measure that approached statistical significance for differences between the SC group and the SN group at Visit A when all of the participants were still HIV-uninfected, and it continued to demonstrate a marginal difference between the two groups at the post-HIV infection Visit B. This suggests that there are factors included in this clock that predict mortality but are not impacted, at least during the first months and years, by changes picked up by other epigenetic measures that occur when an individual first becomes infected with HIV. GEAA was developed utilizing, among other markers, DNA methylation-based surrogate markers related to smoking pack-years. The small but persistent differences in the GEAA clock, with SC showing slightly more age acceleration than SN, is consistent with SC reporting more smoking (greater mean cumulative pack-years) than the SN group, even before HIV infection. It is possible, therefore, that the role of smoking in shaping DNA methylation patterns detected by the GEAA clock is relatively unaffected by the age acceleration indicated by the other epigenetic measures, which are more likely to reflect immune system changes and immunosenescence. This is supported by our observation that smoking history was not a significant contributing factor in mixed models to the age acceleration seen over the course of initial HIV infection in the AAR, PEAA, and EEAA clocks, nor the aaDNAmTL. Additional analyses are in progress, evaluating the risk conferred by accelerated epigenetic aging indicated by these DNAm measures for specific health outcomes and mortality in treated persons living long term with HIV.

The greater history of smoking among the SC group in this study is consistent with previous observations among MACS men, where greater prevalence of heavy alcohol consumption, and to a lesser degree, smoking, high-risk sexual behaviors, and moderate to heavy drug use, were associated with HIV seroconversion (Penkower et al., 1991). Therefore, smoking history could be viewed as a surrogate for greater risk-taking behaviors associated with becoming HIV-infected. Chronic alcohol consumption has been reported to contribute to DNA hypomethylation (Zakhari, 2013), and so could be impacting genome-wide DNAm patterns included in one or more of the epigenetic measures evaluated here. Risk-taking behavior, including smoking, could be contributing to the marginally-significant difference between SC and SN groups observed only in GEAA, as described above. However, any possible epigenetic effects of smoking and other risky behavior(s) in the SC group before HIV infection were not detected by any of the other epigenetic clocks or the estimate of TL, as those measures showed no differences between SC and SN groups at Visit A.

High risk sexual behaviors that are associated with HIV seroconversion carry other health-related risks, such as infection with HCV, HBV, CMV, or other infectious agents, which in turn, could affect the aging process. Because of these concerns, SC and SN participants were matched on chronologic age and time interval between study visits as well as on HCV status, and HBV status was included as a co-variate in the demographic mixed model analyses. Consistent with other reports in homosexual men (Drew et al., 1981; Nerurkar et al., 1987), infection with CMV in these MACS participants was nearly universal at the first visit evaluated, when all men were HIV-uninfected. Therefore, CMV serostatus was not included among potential contributors to the epigenetic changes observed following initial HIV infection. Although it is possible that prevalent CMV infection could be synergizing with incident HIV infection in its effects on epigenetic aging, it was not possible to examine that question in this population. In a recent very small cross-sectional study(Corley et al., 2021), current serious illness with other viral infections such influenza or COVID-19 were similar on some and different on other epigenetic aging measures (PEAA, GrimAge, DNAmTL) compared to untreated persons living with HIV, with critically-ill COVID-19 patients showing the greatest increase in GrimAge. Genome-wide analyses in the same groups suggested that severe COVID19 may have a different global epigenetic “signature” than established HIV infection. Animal and human studies suggest HCV infection may impact genome-wide DNAm patterns as well as methylation states of key cellular pathways involved in carcinogenesis (Domovitz and Gal-Tanamy, 2021). Although it is likely that at least some of the epigenetic changes observed over the course of HIV infection may not be unique among viral infections, the current longitudinal study design offers a rare insight before and after initial infection with a virus that directly infects and impacts immune cells circulating in the blood.

When we did examine other potential contributors to the epigenetic measures at the two visits (Table2), the study visit itself, i.e., simply the differences between Visit A and B, was strongly associated with all of the epigenetic measures except GEAA. This parallels the within-person changes between visits illustrated by Figure 2, where only GEAA failed to reveal differences in the SC group. When the interaction between study visit and HIV serostatus group was analyzed, it became clear that for the EEAA and PEAA clocks plus the estimated aaDNAmTL (all p < 0.001), the association with study visit was because of the changes in the SC group, i.e., initial HIV infection, even when taking other co-variates into account. Active HBV infection and BMI did not associate with any of the epigenetic measures, and as discussed above, it was not surprising that only GEAA was associated with smoking history. This supports and further strengthens our observation that the processes involved in the initial HIV infection event itself appear to be driving accelerated aging as characterized by the EEAA, PEAA, and aaDNAmTL epigenetic measures. We did observe a small effect of race (non-white vs white) on the estimated aaDNAmTL before any of the participants in our substudy became HIV-infected, which is consistent with recent reports of longer measured telomere length in blacks compared to whites(Rewak et al., 2014). There was a highly significant effect of race on GEAA, which appeared to be driven by non-white participants in the SC group, of which there were only 13 out of 102. Combined with the association with smoking history, these analyses further emphasized that we were unable to detect an impact of initial HIV infection on this epigenetic clock among our participants. However, our specific substudy (and the MACS overall) is limited in the number of non-white participants, and therefore, lacks the statistical power to appropriately explore the role of race in epigenetic measures of aging, highlighting the need to investigate the possibility of differences related to race in the context of HIV infection within more diverse populations.

HIV infects CD4 T cells and monocytes/macrophages of the immune system, and its impact on the number of T cells of various subtypes is part of the hallmark of initial HIV infection and, ultimately, its accompanying immunodeficiency(Killian et al., 2004). Therefore, it is possible that HIV-induced changes in T cell numbers or frequencies may be inextricably linked to observed changes in epigenetic measures over the course of initial HIV infection. We examined this question by utilizing direct flow cytometric measures of T cell subsets and developing two additional sets of mixed models, taking into account five representative T cell subsets that gave the best consensus fit across all five epigenetic measures, but also are known to be altered as a result of HIV pathogenesis (total CD4 and CD8, naïve CD4, activated and senescent CD8) (Killian et al., 2004). The numbers and percentages of total and naive CD4 T cells, and senescent CD8 T cells, were significantly associated directly with most of the epigenetic measures except GEAA (Table 4, Table S10), indicating that for all but GEAA, immune system cell changes were playing an important role. However, the analyses of the interaction of study visit and HIV serostatus group, even after taking into account the five different T cell subset numbers or percentages, still showed a significant association with the same three epigenetic measures, EEAA, PEAA, and aaDNAmTL. This is consistent with at least one other study looking at chronic HIV infection using the Hannum Clock (Gross et al., 2016), which found that being infected with HIV had an effect beyond cell composition. Therefore, even though the changes in critical T cell numbers and percentages are strong contributors to the epigenetic changes observed, initial HIV infection is still making an additional contribution to accelerated aging as characterized by these particular epigenetic measures.

It is important to emphasize that this study of early effects of untreated HIV on these epigenetic measures was not designed to, and therefore, cannot directly answer whether such changes will ultimately be sustained and/or can predict longer term clinical and functional outcomes associated with aging such as co-morbidities and frailty, especially after initiation of highly active antiretroviral therapy (HAART). It has been reported that senescent T cell phenotypes(Hunt et al., 2014; Lee et al., 2014; Tenorio et al., 2014) and direct measures of TL(Erlandson et al., 2013) have failed to reveal a link with aging outcomes in persons living with HIV. However, this does not rule out the possibility that, early in HIV infection, some epigenetic measures (which capture different aspects of biological aging than T cell phenotypes alone) may have predictive value. Rather, our fundamental epigenetic observations at the time of infection highlight the need for additional studies (which are currently in progress) to determine whether epigenetic measures of aging continue to be associated with and predict subsequent development of morbidities, mortality, and frailty in persons living with and treated for HIV.

Before the availability of and/or use of HAART, it was shown that newly HIV-infected individuals typically experienced very high plasma HIV VL levels during acute infection, which resolved within the first year to a lower and relatively stable level, known as the HIV viral set-point(Mellors et al., 1996; De Wolf et al., 1997; Lefrère et al., 1998). By design, for the current study, the post-HIV infection time point in the SC was selected to be after the acute infection period, once the set-point had been established, but still within a relatively short time during which epigenetic aging effects would not be expected to be seen in the matched SN. In addition, the post-HIV infection time point was required to be before the initiation of HAART. This enabled us to demonstrate significant positive correlations between HIV VL and the EEAA and PEAA clocks (i.e., older epigenetic age), and negative correlations with the estimated aaDNAmTL (i.e., estimated shortening of telomeres) at the post-HIV infection visit (Figure S1). This is consistent with the mixed models demonstrating a role for initial HIV infection even after controlling for T cell changes, and supports the concept that the amount of HIV present as a result of the viral set-point may contribute to the magnitude of the early acceleration of biological aging according to these three epigenetic measures. It is important to acknowledge, however, that the viral set-point is the cumulative result of the interplay of virologic, immunologic, and genetic factors over the course of the initial period of HIV infection, and not the virus itself acting in isolation. Nonetheless, the possibility of HIV VL early after infection contributing to accelerated biological aging, with predicted risks of earlier mortality and immune system senescence by EEAA and PEAA, and dramatic estimates of shortened telomeres, adds yet another reason to strive to achieve clinical suppression of HIV in as many persons with HIV as possible, as soon as possible after infection.

In addition to the five calculated epigenetic measures reported here, which are based on carefully validated CpGs, this case/control longitudinal study has generated a unique DNAm dataset from 850,00 + CpG sites, and provides an exciting opportunity to explore the epigenetic imprint of initial HIV infection. Our methylome-wide analyses have provided illumination of the many gene networks and pathways associated with HIV seroconversion. Similar to our previous report in persons living with HIV(Rickabaugh et al., 2015), enrichment analyses identified many genes and pathways associated with embryogenesis and morphogenesis. This is biologically consistent with the accelerated aging indicated by many of the epigenetic clocks and our previous published results demonstrating significant overlap of aberrantly methylated genes and gene pathways influenced by both HIV and aging, particularly in the polycomb group target protein pathway (Rickabaugh et al., 2015). In addition, and not too surprisingly, many genes and pathways involved in immune responses were also altered by initial HIV infection. The ability of HAART to normalize these gene pathways and the epigenetic clocks is a critically-important question, but this report focuses on the rare opportunity to examine the question of epigenetic changes from before, to very soon after, documented initial HIV infection, when PBMC sample availability is extremely limited and very precious. A recent report by our group of a preliminary study of 15 pairs of HIV-infected and uninfected MACS participants demonstrated that initiation of HAART may slightly improve epigenetic measures of accelerated aging, but does not return any of them to levels comparable to HIV-uninfected persons of the same chronologic age(Sehl et al., 2020). Similarly, that report utilized WCGNA to identify a cluster of CpG sites impacted by treatment of HIV, paving the way for additional analyses in a larger case-control dataset (manuscript in preparation, Sehl et al.).

Three of the greatest strengths of the MACS/MWCCS are the length of its longitudinal follow-up (from the early 1980s to the present), the depth of its repository of biologic samples (typically collected every 6 months), and its inclusion of both HIV-infected and uninfected participants from the same at-risk population. Coupled with an extensive demographic and clinical database, we are able to utilize the MACS to not only explore critical longitudinal questions related to HIV infection itself, but to address longer term questions related to living and aging with HIV. The extraordinary opportunity to evaluate biologic aging in more than one hundred individuals over the course of initial HIV infection, and in parallel in matched individuals who were documented to remain HIV-uninfected, was only possible in a prospective study like the MACS/MWCCS. Additional longitudinal analyses have very recently demonstrated that, over the course of years living with HIV before beginning HAART, the rate at which epigenetic age increases and estimated TL shortens is two to three times faster in HIV-infected men (Sehl et al., 2022). This emphasizes the importance of recognizing how quickly the process of initial HIV infection begins accelerating epigenetic measures of aging, and lays a foundation for further exploration of characterizing these epigenetic measures as predictors of future clinical outcomes and impacts on healthspan.

## Limitations of the study

The MACS includes only men who have sex with men, limiting the generalizability of our results to women living with HIV. The MACS enrolled small numbers of non-white participants, especially in the early years of the cohort when many of the documented new HIV infections and seroconversions occurred. Although this epigenetic substudy could not be designed with sufficient statistical power to examine both initial HIV infection and race, later enrollments for the MACS, and the merged MWCCS (which includes both men and women at risk or living with HIV) are intended to provide a more diverse cohort for future studies including those focused on aging with HIV(D’souza et al., 2019). Because of the scarcity of viably-preserved pre-HIV seroconversion PBMC samples, our sample size was limited to approximately 100 seroconverters, not all of whom also had post-treatment samples available. Based on our preliminary study(Sehl et al., 2020), a sample size of 200 HIV-infected individuals and matched controls is needed to properly evaluate the impact of initiation of HAART on these epigenetic measures, so our current sample has insufficient power to address this and other important issues such as prediction of clinical outcomes. Flow cytometry was designed to assess T cell subsets known to be important to HIV pathology and immunosenescence, which limited our ability to account for contribution of changes in non-T cell populations to changes in epigenetic measures.

## STAR★Methods

### Key resources table

**Table:** 

REAGENT or RESOURCE	SOURCE	IDENTIFIER
**Antibodies**		

CD3-PerCP	BD Biosciences	SK7 (RUO (GMP)), Cat#347344; RRID: AB_400286
CD4-V450	BD Biosciences	RPA-T4 (RUO), Cat#561838; RRID: AB_10924599
CD8-APC-Cy7	BD Biosciences	SK1 (RUO (GMP)), Cat#348793; RRID: AB_400383
CD45RA-PE-Cy7	BD Biosciences	L48 (RUO (GMP)), Cat#337167; RRID: AB_647424
CCR7-AF647	BD Biosciences	150,503 (RUO), Cat#560816; RRID: AB_2033948
HLA DR-BV605	BD Biosciences	G46-6 (RUO), Cat#562845; RRID: AB_2744478
CD38-PE	BD Biosciences	HB7 (RUO (GMP)), Cat#347687; RRID: AB_400341
CD28-PE	BD Biosciences	L293 (RUO (GMP)), Cat#348047; RRID: AB_400368
CD57-BV605	Biolegend	QA17A04 (RUO), Cat#393304; RRID: AB_2728426
CD4-BV510	Biolegend	OKT4 (RUO), Cat#317444; RRID: AB_2561866
IgG2a-AF647	BD Biosciences	G155-178 (RUO), Cat#557715; RRID: AB_396824
IgG2a-BV605	BD Biosciences	G155-178 (RUO), Cat#562778; RRID: AB_2869434
IgG1-PE	BD Biosciences	×40 (RUO (GMP)), Cat#349043; RRID: AB_400398
IgG1-PE-Cy7	BD Biosciences	MOPC-21 (RUO), Cat#557872; RRID: AB_396914

**Biological samples**		

Viably-frozen peripheral blood mononuclear cells	MACS/WIHS Combined Cohort Study (MWCCS)	MWCCS concept sheet number C15039

**Chemicals, peptides, and recombinant proteins**		

Zombie Aqua Fixable Viability Kit	Biolegend	(RUO), Cat#423102

**Critical commercial assays**		

DNeasy Blood & Tissue Kit	QIAGEN	Cat#69506
Quant-it PicoGreen dsDNA Assay Kit	Invitrogen	Cat#P7589
EZ-96 DNA Methylation Kit	Zymo Research	Cat#D5004
Infinium MethylationEPIC BeadChip	Illumina	Cat#WG-317-1003

**Deposited data**		

Raw methylation data	This paper	MACS/WIHS Combined Cohort Study (MWCCS) when study aims are completed per MWCCS policy, via the concept sheet approval process (https://statepi.jhsph.edu/mwccs/work-with-us/); MWCCS concept sheet number C15039
Calculated age-regressed epigenetic clock and estimated telomere length data, as well as necessary de-identified demographic or descriptive data	This paper	MWCCS upon reasonable request via the concept sheet approval process (https://statepi.jhsph.edu/mwccs/work-with-us/); MWCCS concept sheet number C15039

**Software and algorithms**		

Epigenetic clock software Cell proportion imputation software	http://dnamage.genetics.ucla.edu	Horvath (2013); Hannum et al., 2013; Chen et al., 2016; Levine et al., 2018; Lu, Quach, et al., 2019a; Lu, Seeboth, et al., 2019b; Horvath and Levine (2015); Horvath et al., 2016
Weighted Gene Correlation Network Analysis, WGCNA R package	This paper	Langfelder and Horvath (2008)
EnrichR gene list enhancement tool	This paper	Kuleshov et al. (2016)
Attune NxT Software	ThermoFisher	Cat#A25556

### Resource availability

#### Lead contact

Further information and requests for resources should be directed to and will be fulfilled by the lead contact, Dr. Beth Jamieson (bjamieso@ucla.edu).

#### Materials availability

This study did not generate new unique reagents.

### Experimental model and subject details

#### Human subjects

Participants for the study reported here were selected from among participants of the Multicenter AIDS Cohort Study (MACS), now part of the MACS/WIHS Combined Cohort Study (MWCCS). The MACS is an ongoing prospective study of the natural and treated history of Human Immunodeficiency Virus (HIV) infection in men who have sex with men (Kaslow et al., 1987). Participants for the current substudy of initial HIV infection were selected whenever possible from among MACS participants from a large biomarker study, which has been described elsewhere (Wada et al., 2015). The MWCCS complies with all relevant ethical regulations, including obtaining informed consent for research from all study participants. This MACS/MWCCS substudy was given exempt status by the University of California, Los Angeles Medical Institutional Review Board IRB#15001179.

102 men were selected who had undergone documented initial HIV infection and HIV antibody seroconversion (SC) after entry into the MACS, and 102 matched persistently HIV seronegative (SN) men. Selection criteria are described below in the Method Details section. Clinical and demographic information on all subjects can be found in Table 1.

The original predetermined sample size calculation was as follows: a sample size of 234 (117 seroconverters and 117 seronegative men) achieves over 80% power to detect an R-squared of 0.05 attributed to the 2 independent variables using an F-Test with a significance level (alpha) of 0.05 and adjusted for an additional 3 confounding variables. Upon querying the MACS/MWCCS repository for archived viably-frozen peripheral blood mononuclear cell (PBMC) sample availability within our specified study design criteria (see study design details below), it was only possible to obtain pre/post HIV infection PBMC samples from 102 seroconverters and from equivalent visits in 102 matched seronegative men. No additional pre/post HIV PBMC samples, which are rare even in large cohort studies, were available to us without compromising the study design, so we proceeded with our original study design with the 102 pairs of participants (204 compared to 234, or 87% of the original calculated sample size).

### Method details

#### Participant selection and samples

Viably-frozen peripheral blood mononuclear cells (PBMC) were obtained from the national repository of the MACS/MWCCS. MACS study visits typically occur at 6 months intervals, clinical and questionnaire data are collected, and peripheral blood samples are processed and frozen.

SC participants were selected who had PBMC samples available in the repository from two time periods: (1) up to 1.5 years prior to the first HIV-seropositive study visit (pre-HIV infection, Visit A), and (2) up to 2.5 years after the first seropositive visit (post-HIV infection, Visit B). The pre-HIV PBMC sample was required to be from a visit that was both HIV antibody seronegative and with undetectable plasma HIV RNA. All post-HIV infection PBMC samples were required to be before initiation of HAART; if multiple PBMC samples were available post-HIV infection, the visit closest to 3 years after the pre-HIV visit was selected. 102 SC with 204 PBMC samples were available for inclusion in the current substudy.

Matched persistently HIV seronegative (SN) controls were then selected among MACS participants for each SC. SN were selected matched by age (±2 years) and Hepatitis C Virus (HCV) status (HCV RNA positive/negative) at both visits, as well as by availability of PBMC at two visits (Visits A and B) with comparable time interval between visits (±0.75 years). 102 matched SN controls were identified, but a PBMC sample matched on age and HCV status within a comparable time interval was not available from one control at Visit A (equivalent to pre-HIV infection in the matched SC), yielding 203 SN PBMC samples.

#### Participant demographics and characteristics

HIV serostatus, plasma HIV viral load (VL), absolute CD4 T cell numbers, Hepatitis B Virus (HBV) and cytomegalovirus (CMV) status, and other demographic and clinical data were available from the MWCCS database, and are summarized in Table 1. Where data are missing from the 102 SC or 102 SN, the exact n is shown in Table 1. An estimated date of HIV infection for each SC was calculated utilizing HIV serostatus (HIV antibody and Western Blot) and HIV VL data from all MACS study visits. Date of HIV infection was estimated as the midpoint between the last MACS study visit at which the participant was HIV seronegative and HIV VL undetectable (if VL data were available) and the first MACS study visit with either HIV-positive serostatus or detectable HIV VL, whichever came first. For 10 SC for whom VL data were missing at Visit B, the VL from the MACS visit immediately prior was used (0.3–0.5 years prior, approximately 3–6 months). For post-HIV infection visits with undetectable VL, a value equal to the lower limit of detection of the VL assay was assigned; four SC had VL < 400 copies/mL (Roche Amplicor 2^nd^ generation assay, Roche Molecular Systems, Branchburg, NJ, USA), and 1 had <50 copies/mL (ultra-sensitive Roche Amplicor assay). HBV status at each visit was categorized as positive (HBV surface antigen [HBsAg] positive) or negative (HBsAg negative), and smoking history was evaluated by cumulative pack years reported. CMV status at Visit A (pre-HIV infection visit in SC group, equivalent visit in SN group) was categorized when available as seropositive (detectable anti-CMV antibody titer before or at Visit A) or seronegative (undetectable anti-CMV antibody titer at or after Visit A), based on data in the MWCCS database from testing of selected MACS visits between 1984 and 1989.

#### Thawing and viability of frozen samples

Frozen PBMC vials were removed from liquid nitrogen storage tanks and placed into a 37 °C water bath. Once samples were thawed and removed from the water bath, the vials were wiped down with 70% isopropyl alcohol and the cell suspensions transferred using plastic transfer pipettes (Fisher Brand) to a 15 mL round bottom tube (Corning). Roswell Park Memorial Institute Medium (RPMI [Gibco]) with 10% Fetal Calf Serum (FCS [Omega]), 1% L-glutamine (Gibco), 100 U/mL Penicillin-Streptomycin (Gibco), 12.5 mM 4-(2-hydroxyethyl)-1-piperazineethanesulfonic acid (HEPES [Gibco]) buffer was added dropwise to each round bottom tube to gradually dilute the cell suspension. Between dropwise additions of culture medium, the cell suspensions were agitated to ensure homogeneity. Cells were centrifuged at 300g for 10 min at room temperature, then resuspended in 1 mL of 10% FCS-RPMI. Cell suspensions were counted using a Z2 Particle Counter (Beckman Coulter) and viabilities were assessed using 1:4 dilution of cell suspension to 0.2% Trypan Blue under a hemocytometer chamber; mean viability of PBMC was 89.6%. Thawed PBMC were divided for DNA extraction and flow cytometry.

#### Genomic DNA isolation and quantification

1.0 × 10^6^ viable PBMC were diluted with 1 mL of Phosphate Buffer Solution (PBS [Gibco]) then spun at 2200g in a 5415C centrifuge (Eppendorf) for 3 min in a 2mL conical screw top tube (Stardstedt). Supernatants were aspirated, leaving a dry PBMC pellet which was stored in a −80°C freezer until genomic DNA isolation.

DNeasy Blood and Tissue kits (Qiagen) were used according to kit protocol to extract DNA from frozen dry PBMC pellets. 200 microliters of PBS, then 20 microliters of proteinase K and finally 20 microliters of Buffer AL was added to the dry pellet tubes. Mixtures were vortexed after each addition, then incubated in a 56°C water bath for 5 min. DNA mixtures were vortexed again and placed back into the water bath for an additional 5 min. 200 microliters of 200 proof ethanol was added to the mixture, vortexed, and then added to the DNeasy Mini spin columns. Columns were spun for 1 min at 6000g at room temperature. Flow through and collection tubes were removed and discarded. Spin columns were then placed into a new collection tube, 500 microliters of Buffer AW1 added to each column, and spun at 6000g for 1 min at room temperature. Again, flow through was discarded and columns were put into another collection tube, 500 microliters of Buffer AW2 added and spun at 14000g room temperature for 3 min. Flow through was once again discarded and columns placed into a new collection tube. 200 microliters of Buffer AE was added to the columns, incubated at room temperature for 1 min then centrifuged at 6000g for 1 min. This step was repeated 2 more times to increase DNA yield. 600 microliters DNA suspensions were added to Microcon DNA Fast Flow Centrifgual Filters (Millipore) and 2 mL collection tubes (Fisher Brand). Suspension, filter, and tube units were spun at 400g for 10 min. Centrifugal filters were then inverted into a new collection tube and spun at 1500g for 3 min. DNA concentration was determined using a NanoDrop One (ThermoFisher) using the dsDNA setting and automatic measurements generated from 220–340 nm wavelengths. Because the genomic DNA samples were finally resuspended in Buffer AE from Qiagen kits, the same Buffer AE was used to blank the NanoDrop One to ensure the background was accounted for appropriately. Genomic DNA samples were then stored in −80 °C freezers until plated for methylation analysis.

#### DNA methylation arrays

Methylation status at more than 850,000 potential methylation sites (CpGs) were measured using the Infinium MethylationEPIC BeadChip (Illumina, San Diego, CA), by the UCLA Neuroscience Genomics Core (https://www.semel.ucla.edu/ungc). Blinded matched sets of genomic DNA samples were created by the investigators, with each set containing samples from matched SN and SC participants at all visits; sets were then placed on an 8-well BeadChip by the Genomics Core. DNA concentrations were determined using a Quant-it PicoGreen dsDNA Assay Kit (Invitrogen/Molecular Probes, Inc., Eugene, OR). 500 ng of genomic DNA was bisulfate converted using the EZ-96 DNA Methylation Kit (Zymo Research, Orange, CA), prepared for use in the Illumina Infinium assay, followed by microarray hybridization and scanning (iScan, Illumina), according to the manufacturer’s protocols. The methylation assays routinely included internal laboratory replicates to document satisfactory technical performance. All assays passed the Genomics Core technical requirements. DNA methylation levels (beta values) were determined by calculating the intensity of the methylated (M corresponding to signal A) and un-methylated (U corresponding to signal B) sites, as the ratio of fluorescent signals:

$$\frac{Max\left(M,0\right)}{\left[Max\left(M,0\right)+Max\left(U,0\right)+100\right]}$$

Therefore, beta values range from 0 (completely un-methylated) to 1 (completely methylated). A Euclidean metric (impute.knn function in R) (Troyanskaya et al., 2001) was utilized to find k-nearest neighbors and impute missing beta values by averaging non-missing elements of its neighbors. Quantile normalization was applied to the raw data, to detect and remove outliers, and to make data comparable to the training data of the epigenetic clocks and consistent with previous analyses(Sehl et al., 2020).

#### Flow cytometry for T Cell subsets

Percentages of naive (CD45RA^+^CCR7^+^), activated (HLA-DR^+^CD38^+^), and senescent (CD28^−^CD57^+^) T cells within the total CD4 (CD3^+^CD4^+^) and CD8 (CD3^+^CD8^+^) T cells of each PBMC sample were determined by multicolor flow cytometry (Mahnke et al., 2013; Brenchley et al., 2003). Depending on recovery and viability after thawing, 0.5 × 10^6^ to 1.0 × 10^6^ viable PBMC per tube were stained on the same day as they were thawed for flow cytometry, with a total of 3 staining tubes per sample. PMBCs were surface-stained as shown in Table S13, for 30 min in the dark at room temperature. Specific antibody conjugates were added to each tube (which contained up to 1.0 × 10^6^ cells) in a volume equivalent to one test, as defined by the manufacturer. Isotype antibody volumes added per tube were calculated to match the corresponding markers’ antibody concentrations. The dead cell discriminator, Zombie Aqua, was reconstituted according to manufacturer recommendations: 100 microliters DMSO (Biolegend) was added to each lyophilized reagent, then diluted 1:100 with Phosphate Buffer Solution (PBS, Life Technologies). 100 microliters of the diluted Zombie Aqua was added to each tube to discriminate dead cells. All antibody conjugates were obtained from BD Biosciences with the exception of Zombie Aqua, CD57 Brilliant Violet 605 (BV605), and its corresponding isotype IgG2a (BV605), which were purchased from Biolegend. Conjugates were chosen based on availability and to maximize wavelength absorbance distances between detectors. After the staining incubation period, 1 mL of PBS with 2% newborn calf serum (Life Technologies) and 0.1% sodium azide (Sigma Aldrich) were added to each tube and centrifuged at 300g for 10 min. Supernatants were aspirated, and stained PBMC were then resuspended in 200 microliters PBS-sodium azide buffer and transferred into a 96 well round bottom plate (Falcon). Plated PBMCs were acquired and analyzed on an Attune NxT Flow Cytometer (ThermoFisher A29004 [blue/red/violet6/yellow]) with an Attune NxT Flow Cytometer Autosampler (ThermoFisher 4473928), using the Attune NxT Software (ThermoFisher). For each stained sample, 180 microliters of the 200 microliter total sample volume was acquired, yielding up to approximately 800,000 cell events for analysis. To ensure acquisition reliability and reduce cross contamination between samples, run settings of the autosampler were set to 100 microliters/minute with 2 mixes and 4 rinses between samples and a 1 s delay of recording after the start of each sample acquisition. Single color antibody and Fluorescence Minus One (FMO) techniques were utilized to establish photomultiplier tube (PMT) settings for acquisition and cursor settings for subset data analysis. Isotype antibodies were implemented to detect any non-specific binding to proteins or binding to Fc receptors. UltraComp eBeads Plus Compensation Beads (ThermoFisher) were used in conjunction with 1 test of each corresponding antibody. CD4 BV510 (Biolegend) was implemented to compensate the Violet 2 channel as recommended by the manufacturer since CD4 BV510 has similar emissions to Zombie Aqua. Due to the weight of the beads, the compensation panel was acquired using individual tubes instead of the plate reader. Attune Performance Tracking Beads (ThermoFisher) and chicken red blood cells (Biosure) tracked the Attune’s performance over time and ensured accuracy and sensitivity of the instrument.

### Quantification and statistical analyses

#### Epigenetic age acceleration measures

Five measures of epigenetic age acceleration were estimated for each of the 407 PBMC samples in total, representing samples from all participants at both visits, using the online epigenetic clock software (http://dnamage.genetics.ucla.edu). Each of these DNA methylation-based estimates was calculated using methylation beta values obtained from the Infinium MethylationEPIC BeadChip, on all samples at the same time without linkage to HIV serostatus group. There were no adjustments for multiple comparisons, as each epigenetic measure was developed taking this into account. Features of each clock examined are provided in Table S1. Briefly, Age Acceleration Residual (AAR) is based on the DNAm age estimated from 353 CpGs of Horvath’s original epigenetic clock(Horvath, 2013), which is then regressed on chronologic age. AAR captures epigenetic age acceleration (i.e., older epigenetic or biological age than chronological age), is valid for a wide range of tissue types, and is known to be accelerated in disease states. Extrinsic epigenetic age acceleration (EEAA) is based on 71 CpGs of Hannum(Hannum et al., 2013), and was constructed to be positively correlated with senescent T lymphocytes and negatively correlated with naive T lymphocytes(Chen et al., 2016; Hannum et al., 2013).This measure captures both intrinsic methylation changes and extrinsic blood cell composition changes. Second generation clocks, including Phenotypic Age and Grim Age, were examined as they are much stronger predictors of mortality. Phenotypic Epigenetic Age Acceleration (PEAA), based on 513 CpGs, was developed by regressing a phenotypic measure of mortality risk on CpGs(Levine et al., 2018). Grim Epigenetic Age Acceleration (GEAA), based on 1030 CpGs, was developed by regressing time-to-death on DNAm-based surrogate biomarkers of smoking pack-years and a selection of plasma proteins previously associated with mortality or morbidity (Lu et al., 2019a). Finally, a DNA methylation-based estimator of Telomere Length adjusted for chronologic age (aaDNAmTL) was examined in our analyses to evaluate whether HIV infection causes accelerated shortening of telomeres with increased age and/or rate of cellular replication (Lu et al., 2019b).

In this study, there is one time point occurring post-seroconversion to examine the difference between HIV-infected and uninfected, so it is not possible to determine exactly when and over what period of time the observed elevation in epigenetic age occurs. However, “epigenetic age acceleration” measures are compared, defined as the residual that results from regression of epigenetic age on chronologic age, at both visits for the SC and SN groups. Because these measures are age-adjusted, they are technically termed “accelerations” in the epigenetic clock literature, rather than elevations or advancements, even though the time course and rate of acceleration is unknown.

#### Absolute counts of T Cell subsets

Percentages of naive, activated, and senescent T cells among CD4 T cells, or among CD8 T cells, were directly determined in each aliquot of thawed viable PBMC (see Table S13 for staining protocol); out of 407 thawed aliquots of PBMC utilized in DNAm analyses in SC and SN groups at visits A and B, 404 had sufficient viable PBMC to stain for flow cytometry. Absolute total CD4 and total CD8 T cell counts (cells/mm^3^) for almost all samples were available from the MACS/MWCCS database, which had been determined by standardized protocols on the day each blood sample was originally obtained(Giorgi et al., 1990; Hultin et al., 2007). Flow cytometry percentages on thawed PBMC were utilized in combination with absolute total CD4 and CD8 T cell counts to calculate absolute cell counts for naive, activated, and senescent CD4 and CD8 T cells for each sample. Naive and senescent CD4 and CD8 T cells were stained and analyzed in one tube, and activated CD4 and CD8 T cells were stained and analyzed in a separate tube. Due to technical issues during flow cytometry acquisition, the activated cell tube, or both tubes was/were unable to be acquired on a small number of PBMC samples at one or both visits, and/or the absolute CD4 and CD8 T cell counts were missing from the MWCCS database, resulting in some variability in the number of PBMC samples for which absolute cells counts could be calculated, as shown in Table 3.

#### Frequencies of T Cell subsets within the live lymphocyte population

Percentages of total CD3 T cells, CD4 T cells, and CD8 T cells, and naive, activated, and senescent CD4 and CD8 T cells among the total live lymphocytes in each aliquot of thawed viable PBMC were calculated by utilizing the live lymphocyte gate established by forward vs. side scatter for lymphocytes combined with Zombie Aqua viability dye (lymphocytes negative for Zombie Aqua staining). As noted above, naive and senescent CD4 and CD8 T cells, and activated CD4 and CD8 T cells, were stained and analyzed in separate tubes. Therefore, staining for total CD3 T cells, and for CD4 and CD8 T cell subsets, were performed in duplicate for each PBMC sample. When available, duplicate determinations were utilized to calculate mean percentages of live lymphocytes for CD3, CD4, and CD8 cells. However, mean values with a coefficient of variation (CV) > 10% between the two tubes from the same PBMC sample were excluded. Of 404 PBMC samples with sufficient viable cells to stain for flow cytometry, 27 samples were excluded due to CVs >10% on mean % CD3, and 1 each was excluded due to CVs >10% on mean % CD4 and % CD8. Due to additional technical issues during acquisition as described above, there is some variability in the number of PBMC samples for which percentages were available, as shown in Table S9.

#### Statistical analyses of epigenetic measures

No raw methylation data, nor calculated epigenetic clock or estimated DNAmTL data were excluded from the analyses. HIV serostatus groups (SC and SN) were compared to each other (t-test, 2-sided) on each age-adjusted epigenetic measure at Visit A (all participants HIV-uninfected), and again at Visit B (post-HIV infection in SC, time interval-matched visit in uninfected SN). Median values, and 25^th^-75^th^ percentile and 5^th^-95^th^ percentile ranges, are shown in Figure 1. Within-person changes in age-adjusted epigenetic measures from Visit A to Visit B were calculated for each participant (Visit B value – Visit A value), and SC and SN groups were each evaluated by paired t-tests (2-sided) for differences from zero. Median values and 25^th^-75^th^ percentile ranges are shown in Figure 2. Similar analyses were performed on the absolute counts of T cell subsets (Table 3) and percentages of T cell subsets within live lymphocytes (Table S9), comparing SC vs SN at Visit A and again at Visit B (t-test, 2-sided), as well as for the absolute T cell counts (Table S4), evaluating within-person changes from Visit A to Visit B in SC and SN (t-tests, 2-sided, for differences from zero).

Pairwise correlation analyses were performed for each of the epigenetic measures, for all participants at Visit A (all HIV-uninfected), and for SC at Visit B (recently HIV-infected) and SN at Visit B (persistently uninfected). Pearson correlation coefficients (rho) and p values are shown in Table S3. Pairwise correlation analyses were also performed for each of the absolute T cell subsets counts as well as all of the epigenetic measures, for all participants at Visit A, for SC at Visit B, and for SN at Visit B. Pearson correlation coefficients (rho) and p values are shown in Tables S5–S7.

Potential contributions of co-variates (study visit, HIV serostatus group, interaction between study visit∗HIV serostatus group, race [non-white vs. white], current HBsAg status [negative vs. positive], Body Mass Index [BMI], and smoking cumulative pack years) to the changes in each age-adjusted epigenetic measure between the two visits were analyzed in linear mixed effects models using random intercept and slope, with all participants in the same model. Due to missing data for some demographic co-variates, n = 387 samples for these mixed models. The F values and p values from the mixed effect models for all five epigenetic measures are shown in Table 2; individual parameter estimates and p values from fixed effects analyses are reported in Table S2. We assumed compound symmetry covariance structure as more complicated models did not offer a better fit. The code for these mixed models was as follows:

**PROCMIXED** data = THREE;

CLASS macsidnumber casecontrol visit;

MODEL ***aar*** = visit casecontrol casecontrol∗visit white

hepb

CUM_PKYEAR

BMI/SOLUTION outpred = PREDAAR;

RANDOM INTERCEPT/SUB = macsidnumber TYPE = CS G GCORR;

REPEATED visit;

LSMEANS casecontrol/DIFF = all;

ESTIMATE "Control Mean" intercept 1 casecontrol 1 0;

ESTIMATE "Case Mean" intercept 1 casecontrol 0 1;

LSMEANS casecontrol/DIFF = ALL AT (visit)=(1);

LSMEANS casecontrol/DIFF = ALL AT (visit)=(2);

ESTIMATE "Age Slope for Control" visit 1 casecontrol∗visit 1 0;

ESTIMATE "Age Slope for Case" visit 1 casecontrol∗visit 1 0;

store out = MixedModel***AAR***;

run;

quit;

For each additional DNAm measure, substitute the name ***eeaa***, ***peaa***, ***geaa***, or ***dnamtladjage*** where ***aar*** currently appears.

Potential contributions of absolute counts of T cell subsets (total CD4, total CD8, naive CD4, naive CD8, activated CD4, activated CD8, senescent CD4, senescent CD8) to the changes in each age-adjusted epigenetic measure between the two visits were analyzed in linear mixed effects models using random intercept and slope, with all participants in the same model. All absolute T cell counts were natural log-transformed (ln cells/mm^3^) before inclusion into mixed models. Due to missing data for some flow cytometry variables, n = 374 samples for these mixed models. Five potential models were evaluated for all five epigenetic measures. Each model was constructed with a different combination of 3–5 T cell subsets, based on expected HIV pathogenesis and/or to minimize highly-correlated subsets (Tables S5–S7) to reduce co-linearity. We selected the model with the consensus best fit across all five epigenetic measures using the Akaike Information Criterion (AIC); this model included: total CD4, total CD8, naive CD4, activated CD8, senescent CD8. The F values and p values from the mixed effect models for all five epigenetic measures are shown in Table 4; individual parameter estimates and p values from fixed effects analyses are reported in Table S8. The code for these mixed models was as follows:

PROC MIXED data = THREE;

CLASS macsidnumber casecontrol visit;

MODEL ***aar*** = visit casecontrol casecontrol∗visit lnabs_cd42

lnabs_cd82

lnAbsNaiveCD4

lnAbsSenCD8

lnAbsActCD8/SOLUTION outpred = PREDAAR;

RANDOM INTERCEPT/SUB = macsidnumber TYPE = CS G GCORR;

REPEATED visit;

LSMEANS casecontrol/DIFF = all;

ESTIMATE "Control Mean" intercept 1 casecontrol 1 0;

ESTIMATE "Case Mean" intercept 1 casecontrol 0 1;

LSMEANS casecontrol/DIFF = ALL AT (visit)=(1); ∗ group intercept diffs;

LSMEANS casecontrol/DIFF = ALL AT (visit)=(2); ∗ group intercept diffs;

ESTIMATE "Age Slope for Control" visit 1 casecontrol∗visit 1 0;

ESTIMATE "Age Slope for Case" visit 1 casecontrol∗visit 1 0;

store out = MixedModel***AAR***;

**run**;

**quit**;

For each additional DNAm measure, substitute the name ***eeaa***, ***peaa***, ***geaa***, or ***dnamtladjage*** where ***aar*** currently appears.

The consensus best fit model was repeated for each of the DNAm measures to account for natural log-transformed T cell subset percentages instead of absolute T cell counts, using the same code, substituting:

lncd4 for lnabs_cd42

lncd8 for lnabs_cd82

lnNaiveCD4 for lnAbsNaiveCD4

lnSenCD8 for lnAbsSenCD8

lnActCD8 for lnAbsActCD8.

Due to missing data for some flow cytometry variables, n = 382 samples for these mixed models. The F values and p values from these mixed effect models for all five epigenetic measures are shown in Table S10.

At the post HIV-infection visit (Visit B) in the SC group only, regression analyses were performed for each of the epigenetic measures and HIV VL, as well as for HIV VL and absolute CD4 T cell counts together in a single analysis. In all analyses where HIV VL was included, viral load (copies/mL) was log10 transformed. For the analyses with HIV VL, Pearson correlation coefficients (rho) and p values are shown in Figures S1A–S1E.

#### Weighted Gene Correlation Network Analysis (WGCNA) of genomic methylation data

Weighted Gene Correlation Network Analysis (WGCNA) (Langfelder and Horvath, 2008) was utilized to identify clusters of CpGs that are correlated with each other across the genomic DNA (co-methylation modules) of the samples analyzed (all samples from all participants at both visits, n = 407 samples), using methylation levels measured at over 850,000 individual CpG methylation sites on the Infinium MethylationEPIC BeadChip. Missing values were imputed using k-nearest neighbor imputation implemented in function impute.knn in R package impute (Troyanskaya et al., 2001). CpGs were then sorted by decreasing variance and the top 400,000 CpGs were retained for WGCNA. Because network analysis on 400,000 CpGs in a single block is impractical, pre-clustering implemented in WGCNA R package was used to split the CpGs into blocks of no more than 40,000 variables. Network construction and module identification was then carried out in each block separately. Average linkage hierarchical clustering was performed using the topological overlap-based dissimilarity measure, and modules, defined as branches of the resulting clustering tree, were identified using the Dynamic Hybrid branch cutting approach implemented in the R package dynamicTreeCut. A total of 67 co-methylation modules were identified by WGCNA. A representative methylation profile for each module, referred to as the module eigenvector, was defined as the first principal component of the module methylation matrix, and for each CpG within each module, the intramodular connectivity measure kME was calculated as

$$kME_{i}^{I}=cor\left(x_{i},E^{I}\right)$$

where *x*_*i*_ is the methylation profile of CpG labeled *i* and *E*^*I*^ is the representative of module *I*. kME can be considered a continuous measure of module membership for each CpG(Horvath and Dong, 2008). When a CpG has a high kME for a given module (e.g., above a threshold value ≥ 0.85), it is considered a hub site.

Mean and standard deviation (SD) eigenvector methylation values for each Module at each Visit were calculated separately on 102 HIV seroconverters (SC group), and on 101 persistently HIV-uninfected controls (SN group), who had observations at both Visits A and B. Non-parametric group comparison tests (Kruskal-Wallis) were performed in each group, comparing mean Module eigenvector methylation from Visit A to Visit B. The level of significance for changes in mean Module eigenvector methylation values adjusting for multiple comparisons was p < 0.05/67 or <7.5 × 10^−4^. In the SC group, out of the 67 Modules, there were 18 Modules with a p value < 7.5 × 10^−4^, indicating a significant association between changes in methylation values among CpGs in each of these Modules and initial HIV infection (i.e., a change from HIV-uninfected at visit A to HIV-infected at visit B). These 18 Modules are summarized in Table 5. None of the 67 Modules showed significant differences between visits A and B in the SN group, and were not analyzed further.

All of the CpGs from each of the 18 HIV infection-associated Modules are listed in Table S11, which is a supplemental Excel file. The file lists, on a separate tab for each Module, the CpG Illumina ID number for the unique site on the EPIC Bead Chip, name(s) of the gene(s) that contain(s) the unique CpG site, and the kME value for each CpG. The gene names are derived from the Illumina array UCSC_RefGene_Name, which is the target gene name from the UCSCdatabase. If a CpG site falls within multiple genes, all gene names are shown separated by semi-colon. Multiple listings of the same gene name indicate splice variants.

#### Pathways enrichment analyses of genomic methylation data

Enrichment analyses were performed using the EnrichR gene list enhancement tool(Kuleshov et al., 2016) to identify overrepresented biological pathways for those CpG sites with high connectivity (kME ≥0.85 from WGCNA) within each of the 18 HIV infection-associated Modules. Table S12 (a supplemental Excel file) lists, on a separate tab for each Module, enrichment terms (names of biological processes or pathways) identified in the EnrichR analysis, along with the overlap (number of genes identified in our analysis over the total number of genes in the literature for a given pathway), p value (computed using Fisher exact test, assuming a binomial distribution and independence for probability of any gene belonging to any set), adjusted p value (correction for all known genes in the set), Odds Ratio for each enrichment term [given by (a/b)/(c/d), where a = number of genes in the Module that fall in the enrichment term, b = number of genes in the Module that do not fall in the enrichment term, c = number of genes in the enrichment term that are not in the Module, and d = 20,000-(a+b + c); 20,000 is the total number of genes in the human genome], and combined score (the product of the log of the p value from the Fisher exact test with the *Z* score deviation from the expected rank for each term in each gene-set library) for each enrichment term identified for each Module. Enrichment terms within each Module are ordered based on p value, from most to least significant.

### Additional resources

N/A

## Data Availability

• The raw Infinium MethylationEPIC BeadChip methylation data that support the findings reported in this study cannot be deposited in a public repository at this time because of the policies of the MACS/WIHS Combined Cohort Study (MWCCS) from which they were generated. Per MWCCS policies, these raw data will be released via a concept sheet approval process (https://statepi.jhsph.edu/mwccs/work-with-us/) once the original aims of our approved study are complete. All analytic data utilized in this paper (calculated age-regressed epigenetic clock and estimated telomere length data, T cell counts and percentages, as well as necessary de-identified demographic or descriptive data), have been deposited with the MWCCS, and are available upon reasonable request via the MWCCS concept sheet approval process (https://statepi.jhsph.edu/mwccs/work-with-us/). The MWCCS concept sheet number for all data related to this paper is listed in the Key Resources Table. • All original code is available in this paper’s Quantification and statistical analyses section below • Any additional information required to reanalyze the data reported in this paper, if not restricted to the MWCCS concept sheet process, is available from the lead contact upon request.

## Supplemental information

Document S1. Tables S1–S13 and Figure S1

Table S11: WGCNA Results, listing all CpGs in 18 Modules significantly associated with initial HIV infection in SC group, related to Table 5

Table S12: Enrichment Analyses in Modules 1–18 for biological pathways containing genes with at least one CpG with kME ≥0.85 in WGCNA, related to Table 5
