# Accelerated aging with HIV begins at the time of initial HIV infection

**DOI:** 10.1016/j.isci.2023.107381

**Published:** 2023-07-14

**Authors:** Elizabeth Crabb Breen, Mary E. Sehl, Roger Shih, Peter Langfelder, Ruibin Wang, Steve Horvath, Jay H. Bream, Priya Duggal, Jeremy Martinson, Steven M. Wolinsky, Otoniel Martínez-Maza, Christina M. Ramirez, Beth D. Jamieson

## Main text

(iScience *25*, 104488; July 15, 2022)

After publication, the authors became aware of a misclassification error in a portion of the dataset utilized in this article. The original dataset is now replaced by the corrected dataset and is available by request as described in the article. The main conclusions of the article remain valid, but the authors decided to revise the title and summary slightly to better represent the findings. The title has been changed from: “Accelerated aging with HIV begins at the time of initial HIV infection” to “Accelerated epigenetic aging with HIV occurs at the time of initial HIV infection.” The original article and supplemental material are now replaced by the corrected article and supplemental material. The authors apologize for any confusion caused to the readers.

