# Supplementary material for: Accelerated aging with HIV begins at the time of initial HIV infection
Source: iScience. 2022 Jun 30;25(7):104488. doi: 10.1016/j.isci.2022.104488 (PMC9308149; doi:10.1016/j.isci.2022.104488)

iScience, Volume *25*

Supplemental information

Accelerated aging with HIV begins

at the time of initial HIV infection

Elizabeth Crabb Breen, Mary E. Sehl, Roger Shih, Peter Langfelder, Ruibin Wang, Steve Horvath, Jay H. Bream, Priya Duggal, Jeremy Martinson, Steven M. Wolinsky, Otoniel Martínez-Maza, Christina M. Ramirez, and Beth D. Jamieson

Table S1: Features of Epigenetic Measures of Biologic Aging that apply to the Illumina 450 and EPIC array platforms, related to Figures 1 and 2, Tables 2 and 4

**Epigenetic measure**	Pan-tissue age clock (Horvath)	Extrinsic age clock	Phenotypic age clock	Grim age clock	DNA methylation-based estimate of telomere length
**Age-adjusted measure used in analyses**	**Age Acceleration Residual (AAR)**	**Extrinsic Epigenetic Age Acceleration (EEAA)**	**Phenotypic Epigenetic Age Acceleration (PEAA)**	**Grim Epigenetic Age Acceleration (GEAA)**	**Age-adjusted DNAmTL (aaDNAmTL)**
**Number of CpGs**	353	71	513	1030	140
**Tissue(s) in which the measure was developed**	Many human tissues and cell types	Peripheral blood	Peripheral blood	Peripheral blood	Leukocytes, adipose, liver, monocytes
**Methodology of development**	Estimator of chronologic age developed on the basis of a wide spectrum of human tissues and cell types. It applies to all tissues and nucleated cell types.	Estimator of chronologic age for blood methylation data. It is defined as a weighted average of the Hannum clock and 3 cell types known to change with age: naïve and senescent T cells, and plasmablasts.	Methylation based estimator of "phenotypic age" which was defined as a linear combination of several clinical parameters. While it is less predictive of chronologic age than the pan tissue clock, it is more predictive of mortality risk and many age related conditions.	Methylation based predictor of mortality risk, developed as an estimator of log transformed hazard ratio from Cox regression model analysis of time-to-death. Covariates include chronologic age, sex, and methylation based surrogate biomarkers of smoking pack-years and plasma proteins.	Methylation based estimator of leukocyte telomere length. It correlates negatively (i.e., shorter telomere length) with increasing age and obesity in blood and other tissues. Its correlation to actual telomere length is relatively weak (r=0.35).
**Special features**	Widely used in epidemiologic studies. Accelerated in HIV, obesity, neurodegenerative disease, and many diseases states. Weakly predictive of mortality risk. Weak association with blood cell composition. Not related to smoking.	Captures both cell-intrinsic methylation changes and age-related changes in blood cell composition. Strongly correlated with blood cell counts: positively correlated with senescent T lymphocyte and negative correlated with naïve T lymphocyte counts. Reflects aspects of immunosenescence, underlying age-related decline in the protective immune response.	Strongly predictive of healthspan and lifespan. Correlated with multi-morbidity, frailty. Correlated with smoking and markers of immunosenescence.	Strong predictor of mortality risk. Predictive of time to cancer. Associated with age at menopause, frailty, heart disease, metabolic syndrome, fatty liver. Correlated with markers of immunosenescence. Statistical analysis needs to adjust for chronologic age and sex as these variables are in the definition.	Reflects cell replicative history. Useful marker of age-related pathologies. Outperforms measured leukocyte telomere length in predicting time to death, time to coronary heart disease, time to congestive heart failure, and association with smoking history. Associated with physical functioning, dietary variables, education and income.

Table S2: Individual parameter estimates and *p* values from mixed effects models incorporating demographic factors for all five epigenetic measures, related to Table 2

		AAR		EEAA		PEAA		GEAA		aaDNAmTL	
**Co-variate**	**Category**	**Estimate (SE)[a], years**	***p* values[b]**	**Estimate (SE), years**	***p* values**	**Estimate (SE), years**	***p* values**	**Estimate (SE), years**	***p* values**	**Estimate (SE), relative units**	***p* values**
Intercept	n/a	2.2 (2.3)	*0.35*	0.1 (3.2)	*0.97*	-1.5 (3.3)	*0.65*	-2.7 (1.6)	*0.09*	0.159 (0.118)	*0.18*
Study Visit	Visit A	-0.7 (0.5)	*0.15*	-0.3 (0.6)	*0.58*	0.3 (0.7)	*0.70*	0.2 (0.2)	*0.36*	0.011 (0.022)	*0.62*
	Visit B	0	.	0	.	0	.	0	.	0	.
HIV Serostatus Group[c]	SC	1.4 (0.7)	*0.07*	4.5 (1.0)	***<0.001***	5.5 (1.1)	***<0.001***	0.9 (0.5)	*0.08*	-0.261 (0.038)	***<0.001***
	SN	0	.	0	.	0	.	0	.	0	.
HIV Serostatus Group* Study Visit	SC*Visit A	-0.9 (0.7)	*0.20*	-4.7 (0.9)	***<0.001***	-4.9 (0.9)	***<0.001***	0.0 (0.3)	*0.96*	0.254 (0.032)	***<0.001***
	SC*Visit B	0	.	0	.	0	.	0	.	0	.
	SN*Visit A	0	.	0	.	0	.	0	.	0	.
	SN*Visit B	0	.	0	.	0	.	0	.	0	.
Race	non-white	0.5 (0.8)	*0.55*	1.5 (1.2)	*0.19*	2.3 (1.2)	*0.07*	3.2 (0.6)	***<0.001***	0.093 (0.044)	***0.03***
	white	0	.	0	.	0	.	0	.	0	.
Hepatitis B Status, HBsAg	negative	-2.6 (2.3)	*0.27*	-1.4 (3.2)	*0.66*	-1.2 (3.3)	*0.71*	0.5 (1.6)	*0.75*	-0.100 (0.119)	*0.40*
	positive	0	.	0	.	0	.	0	.	0	.
BMI, kg/m^2	n/a	0.0 (0.01)	*0.89*	0.0 (0.01)	*0.68*	0.0 (0.01)	*0.75*	0.0 (0.0)	*0.38*	0.000 (0.000)	*0.70*
Smoking, cumulative pack years	n/a	0.0 (0.02)	*0.93*	0.0 (0.03)	*0.87*	0.0 (0.03)	*0.18*	0.1 (0.01)	***<0.001***	-0.001 (0.001)	*0.22*

AAR = Age-Acceleration Residual, EEAA = Extrinsic Epigenetic Age Acceleration, PEAA = Phenotypic Epigenetic Age Acceleration, GEAA = Grim Epigenetic Age Acceleration, aaDNAmTL = age-adjusted DNA methylation-based estimate of telomere length, HBsAg = Hepatitis B surface antigen, BMI = body mass index

a: parameter estimate and standard error (SE) from mixed models for each co-variate; degrees of freedom=188 except for Study Visit=191
b: Pr>t p values from mixed models for differences from zero for each co-variate, while holding all other co-variates fixed; p values in italics, bold if <0.05
c: HIV serostatus groups classified as SC (became HIV infected and seroconverted between Visits A and B) vs SN (persistently HIV uninfected and seronegative at Visits A and B)

Table S3: Pairwise correlations of epigenetic clocks (AAR, EEAA, PEAA, GEAA) and estimated telomere length (aaDNAmTL) with each other, among all participants at Visit A (all HIV-uninfected), SC at Visit B (recently HIV-infected), and SN at Visit B (persistently HIV-uninfected); related to Table 2

	**Pearson Correlation Coefficients (*p values*[a])**				
**Visit A, All** (n=203)	**AAR**	**EEAA**	**PEAA**	**GEAA**	**aaDNAmTL**
**AAR**	1.00	0.61 (***<0.001***)	0.58 (***<0.001***)	0.21 (***0.003***)	- 0.45 (***<0.001***)
**EEAA**	---	1.00	0.72 (***<0.001***)	0.34 (***<0.001***)	-0.69 (***<0.001***)
**PEAA**	---	---	1.00	0.55 (***<0.001***)	-0.64 (***<0.001***)
**GEAA**	---	---	---	1.00	-0.28 (***<0.001***)
**aaDNAmTL**	---	---	---	---	1.00
**Visit B, SC** (n=102)	**AAR**	**EEAA**	**PEAA**	**GEAA**	**aaDNAmTL**
**AAR**	1.00	0.67 (***<0.001***)	0.59 (***<0.001***)	0.11 (*0.28*)	-0.53 (***<0.001***)
**EEAA**	---	1.00	0.74 (***<0.001***)	0.35 (***<0.001***)	-0.71 (***<0.001***)
**PEAA**	---	---	1.00	0.44 (***<0.001***)	-0.66 (***<0.001***)
**GEAA**	---	---	---	1.00	-0.25 (***0.01***)
**aaDNAmTL**	---	---	---	---	1.00
**Visit B, SN** (n=102)	**AAR**	**EEAA**	**PEAA**	**GEAA**	**aaDNAmTL**
**AAR**	1.00	0.66 (***<0.001***)	0.65 (***<0.001***)	0.14 (*0.17*)	-0.52 (***<0.001***)
**EEAA**	---	1.00	0.69 (***<0.001***)	0.18 (*0.07*)	-0.70 (***<0.001***)
**PEAA**	---	---	1.00	0.48 (***<0.001***)	-0.62 (***<0.001***)
**GEAA**	---	---	---	1.00	-0.18 (*0.08*)
**aaDNAmTL**	---	---	---	---	1.00

AAR = Age-Acceleration Residual, EEAA = Extrinsic Epigenetic Age Acceleration, PEAA = Phenotypic Epigenetic Age Acceleration, GEAA = Grim Epigenetic Age Acceleration, aaDNAmTL = age-adjusted DNA methylation-based estimate of telomere length, SC = HIV seroconverter group, SN = HIV seronegative group

a: p values for pairwise correlations in italics, bold if <0.05

Table S4: Mean within-person change in absolute T cell counts, SC group vs SN group, Visit B-Visit A, related to Table 3

	Within-person change in absolute T cell counts, Visit B - Visit A					
	SC			SN		
**T Cell Population**[a]	Mean (SE), n	Median (IQR), n	***p value***[b]	Mean (SE), n	Median (IQR), n	***p value***[b]
CD4 T cells, cells/mm^3	-478 (381) n = 92	-473 (451) n = 92	***< 0.001***	-1 (319) n = 89	23 (375) n = 89	*0.987*
CD8 T cells, cells/mm^3	256 (341) n = 92	212 (424) n = 92	***< 0.001***	12 (232) n = 89	5 (273) n = 89	*0.625*
Naïve (CD45RA$^+$CCR7$^+$) CD4 T cells, cells/mm^3	-162 (174) n = 91	-132 (254) n = 91	***< 0.001***	-31 (195) n = 87	-11 (143) n = 87	*0.145*
Naïve (CD45RA$^+$CCR7$^+$) CD8 T cells, cells/mm^3	-76 (97) n = 91	-59 (118) n = 91	***< 0.001***	-7 (97) n = 87	-3 (90) n = 87	*0.482*
Activated (HLA-DR$^+$CD38$^+$) CD4 T cells, cells/mm^3	3 (16) n = 89	2 (17) n = 89	***0.046***	2 (14) n = 86	0 (14) n = 86	*0.137*
Activated (HLA-DR$^+$CD38$^+$) CD8 T cells, cells/mm^3	154 (147) n = 89	108 (140) n = 89	***< 0.001***	5 (28) n = 86	1 (13) n = 86	*0.097*
Senescent (CD28$^-$CD57$^+$) CD4 T cells, cells/mm^3	-4 (45) n = 91	-3 (19) n = 91	*0.346*	2 (24) n = 87	0 (16) n = 87	*0.436*
Senescent (CD28$^-$CD57$^+$) CD8 T cells, cells/mm^3	32 (87) n = 91	26 (75) n = 91	***0.001***	3 (68) n = 87	-1 (48) n = 87	*0.731*

a: absolute CD4 and CD8 T cell counts obtained from MWCCS database, and were determined by standardized flow cytometry at the time of original blood sample collection; T cell subsets determined by multicolor flow cytometry at the time of thawing of viable PBMC aliquots as described in the STAR Methods, and absolute T cell subset counts calculated from total CD4 and CD8 counts

b: p values from t-test for change within each participant group for differences from zero; p values in italics, bold if <0.05

Table S5: Pairwise correlations of absolute T cell counts to each other, and to each of the epigenetic clocks (AAR, EEAA, PEAA, GEAA) and estimated telomere length (aaDNAmTL), among all participants at Visit A (all HIV-uninfected), related to Table 3

	**Pearson Correlation Coefficients**<br>***p values***[a]<br>**number of observations**												
**Visit A, All**	**CD4**	**CD8**	**Naïve CD4**	**Naïve CD8**	**Activated CD4**	**Activated CD8**	**Senescent CD4**	**Senescent CD8**	**AAR**	**EEAA**	**PEAA**	**GEAA**	**aaDNA mTL**
**CD4**	1.00	0.07<br>*0.34*<br>202	0.78<br>***<0.001***<br>201	0.48<br>***<0.001***<br>201	0.44<br>***<0.001***<br>198	-0.28<br>***<0.001***<br>198	0.26<br>***<0.001***<br>201	0.12<br>*0.09*<br>201	-0.020<br>*0.78*<br>202	-0.17<br>***0.01***<br>202	-0.12<br>*0.10*<br>202	-0.07<br>*0.32*<br>202	0.05<br>*0.48*<br>202
**CD8**	---	1.00	0.03<br>*0.65*<br>201	0.18<br>***0.01***<br>201	0.43<br>***<0.001***<br>198	0.73<br>***<0.001***<br>198	0.30<br>***<0.001***<br>201	0.73<br>***<0.001***<br>201	0.13<br>*0.07*<br>202	-0.07<br>*0.35*<br>202	-0.02<br>*0.83*<br>202	0.11<br>*0.13*<br>202	-0.09<br>*0.21*<br>202
**Naïve CD4**	---	---	1.00	0.60<br>***<0.001***<br>201	0.32<br>***<0.001***<br>198	-0.21<br>***0.003***<br>198	-0.03<br>*0.72*<br>201	0.07<br>*0.35*<br>201	-0.14<br>***0.04***<br>201	-0.34<br>***<0.001***<br>201	-0.30<br>***<0.001***<br>201	-0.16<br>***0.02***<br>201	0.27<br>***<0.001***<br>201
**Naïve CD8**	---	---	---	1.00	0.05<br>*0.46*<br>198	-0.21<br>***0.003***<br>198	-0.09<br>*0.19*<br>201	0.04<br>*0.60*<br>201	-0.13<br>*0.07*<br>201	-0.38<br>***<0.001***<br>201	-0.30<br>***<0.001***<br>201	-0.08<br>*0.24*<br>201	0.39<br>***<0.001***<br>201
**Activated CD4**	---	---	---	---	1.00	0.43<br>***<0.001***<br>198	0.44<br>***<0.001***<br>198	0.29<br>***<0.001***<br>198	-0.04<br>*0.56*<br>198	-0.09<br>*0.19*<br>198	-0.076<br>*0.29*<br>198	-0.02<br>*0.82*<br>198	-0.03<br>*0.69*<br>198
**Activated CD8**	---	---	---	---	---	1.00	0.18<br>***0.01***<br>198	0.39<br>***<0.001***<br>198	-0.008<br>*0.91*<br>198	-0.05<br>*0.45*<br>198	-0.02<br>*0.80*<br>198	0.10<br>*0.16*<br>198	-0.03<br>*0.67*<br>198
**Senescent CD4**	---	---	---	---	---	---	1.00	0.34<br>***<0.001***<br>201	0.18<br>***0.01***<br>201	0.03<br>*0.66*<br>201	0.08<br>*0.23*<br>201	0.05<br>*0.48*<br>201	-0.19<br>***0.01***<br>201
**Senescent CD8**	---	---	---	---	---	---	---	1.00	0.23<br>***<0.001***<br>201	0.05<br>*0.48*<br>201	0.005<br>*0.94*<br>201	0.005<br>*0.94*<br>201	-0.18<br>***0.01***<br>201

AAR = Age-Acceleration Residual, EEAA = Extrinsic Epigenetic Age Acceleration, PEAA = Phenotypic Epigenetic Age Acceleration, GEAA = Grim Epigenetic Age Acceleration, aaDNAmTL = age-adjusted DNA methylation-based estimate of telomere length

a: p values for pairwise correlations in italics, bold if <0.05

Table S6: Pairwise correlations of absolute T cell counts to each other, and to each of the epigenetic clocks (AAR, EEAA, PEAA, GEAA) and estimated telomere length (aaDNAmTL), among SC participants at Visit B (recently HIV-infected), related to Table 3

	**Pearson Correlation Coefficients**<br>***p values***[a]<br>**number of observations**												
**Visit B, SC**	**CD4**	**CD8**	**Naïve CD4**	**Naïve CD8**	**Activated CD4**	**Activated CD8**	**Senescent CD4**	**Senescent CD8**	**AAR**	**EEAA**	**PEAA**	**GEAA**	**aaDNA mTL**
**CD4**	1.00	0.52<br>***<0.001***<br>92	0.66<br>***<0.001***<br>91	0.40<br>***<0.001***<br>91	0.50<br>***<0.001***<br>89	-0.02<br>*0.85*<br>89	0.42<br>***<0.001***<br>91	0.21<br>***0.05***<br>91	0.17<br>*0.10*<br>92	0.10<br>*0.33*<br>92	0.07<br>*0.53*<br>92	0.14<br>*0.19*<br>92	-0.16<br>*0.13*<br>92
**CD8**	---	1.00	0.36<br>***<0.001***<br>91	0.57<br>***<0.001***<br>91	0.14<br>*0.20*<br>89	0.38<br>***<0.001***<br>89	0.47<br>***<0.001***<br>91	0.62<br>***<0.001***<br>91	0.26<br>***0.01***<br>92	0.11<br>*0.28*<br>92	0.13<br>*0.20*<br>92	0.02<br>*0.87*<br>92	-0.17<br>*0.11*<br>92
**Naïve CD4**	---	---	1.00	0.58<br>***<0.001***<br>91	0.29<br>***0.006***<br>89	-0.05<br>*0.64*<br>89	0.05<br>*0.61*<br>91	0.18<br>*0.09*<br>91	-0.01<br>*0.92*<br>91	-0.18<br>*0.09*<br>91	-0.08<br>*0.45*<br>91	-0.02<br>*0.84*<br>91	0.03<br>*0.75*<br>91
**Naïve CD8**	---	---	---	1.00	0.09<br>*0.40*<br>89	-0.03<br>*0.81*<br>89	0.20<br>*0.06*<br>91	0.10<br>*0.35*<br>91	0.10<br>*0.35*<br>91	-0.13<br>*0.21*<br>91	-0.10<br>*0.35*<br>91	-0.10<br>*0.35*<br>91	0.18<br>*0.09*<br>91
**Activated CD4**	---	---	---	---	1.00	0.39<br>***<0.001***<br>89	0.25<br>***0.02***<br>89	-0.05<br>*0.67*<br>89	0.03<br>*0.78*<br>89	0.093<br>*0.38*<br>89	0.033<br>*0.76*<br>89	0.02<br>*0.89*<br>89	-0.08<br>*0.48*<br>89
**Activated CD8**	---	---	---	---	---	1.00	0.13<br>*0.23*<br>89	0.26<br>***0.01***<br>89	0.01<br>*0.90*<br>89	0.02<br>*0.87*<br>89	0.026<br>*0.81*<br>89	-0.06<br>*0.59*<br>89	-0.05<br>*0.64*<br>89
**Senescent CD4**	---	---	---	---	---	---	1.00	0.31<br>***0.003***<br>91	0.28<br>***0.007***<br>91	0.19<br>*0.07*<br>91	0.06<br>*0.57*<br>91	0.05<br>*0.63*<br>91	-0.19<br>*0.08*<br>91
**Senescent CD8**	---	---	---	---	---	---	---	1.00	0.24<br>***0.02***<br>91	0.20<br>*0.06*<br>91	0.16<br>*0.13*<br>91	0.08<br>*0.48*<br>91	-0.23<br>***0.03***<br>91

AAR = Age-Acceleration Residual, EEAA = Extrinsic Epigenetic Age Acceleration, PEAA = Phenotypic Epigenetic Age Acceleration, GEAA = Grim Epigenetic Age Acceleration, aaDNAmTL = age-adjusted DNA methylation-based estimate of telomere length, SC = HIV seroconverter group

a: p values for pairwise correlations in italics, bold if <0.05

Table S7: Pairwise correlations of absolute T cell counts to each other, and to each of the epigenetic clocks (AAR, EEAA, PEAA, GEAA) and estimated telomere length (aaDNAmTL), among SN participants at Visit B (persistently HIV-uninfected), related to Table 3

	**Pearson Correlation Coefficients**<br>***p values***[a]<br>**number of observations**												
**Visit B, SN**	**CD4**	**CD8**	**Naïve CD4**	**Naïve CD8**	**Activated CD4**	**Activated CD8**	**Senescent CD4**	**Senescent CD8**	**AAR**	**EEAA**	**PEAA**	**GEAA**	**aaDNA mTL**
**CD4**	1.00	0.42<br>***<0.001***<br>90	0.82<br>***<0.001***<br>89	0.49<br>***<0.001***<br>89	0.73<br>***<0.001***<br>87	0.17<br>*0.13*<br>87	0.18<br>*0.09*<br>89	0.23<br>***0.03***<br>89	0.03<br>*0.78*<br>90	-0.12<br>*0.25*<br>90	-0.16<br>*0.14*<br>90	0.02<br>*0.85*<br>90	0.04<br>*0.68*<br>90
**CD8**	---	1.00	0.21<br>*0.05*<br>89	0.46<br>***<0.001***<br>89	0.47<br>***<0.001***<br>87	0.69<br>***<0.001***<br>87	0.51<br>***<0.001***<br>89	0.77<br>***<0.001***<br>89	0.28<br>***0.007***<br>90	0.15<br>*0.16*<br>90	0.06<br>*0.56*<br>90	0.09<br>*0.42*<br>90	-0.35<br>***<0.001***<br>90
**Naïve CD4**	---	---	1.00	0.58<br>***<0.001***<br>89	0.70<br>***<0.001***<br>87	0.06<br>*0.55*<br>87	-0.08<br>*0.45*<br>89	0.06<br>*0.59*<br>89	-0.14<br>*0.20*<br>89	-0.33<br>***0.002***<br>89	-0.33<br>***0.002***<br>89	-0.11<br>*0.29*<br>89	0.26<br>***0.02***<br>89
**Naïve CD8**	---	---	---	1.00	0.44<br>***<0.001***<br>87	0.13<br>*0.24*<br>87	-0.11<br>*0.32*<br>89	-0.02<br>*0.86*<br>89	-0.13<br>*0.24*<br>89	-0.46<br>***<0.001***<br>89	-0.36<br>***<0.001***<br>89	-0.06<br>*0.57*<br>89	0.39<br>***<0.001***<br>89
**Activated CD4**	---	---	---	---	1.00	0.53<br>***<0.001***<br>87	0.18<br>*0.10*<br>87	0.31<br>***0.003***<br>87	0.03<br>*0.81*<br>87	-0.12<br>*0.28*<br>87	-0.19<br>*0.08*<br>87	-0.11<br>*0.32*<br>87	0.05<br>*0.62*<br>87
**Activated CD8**	---	---	---	---	---	1.00	0.36<br>***<0.001***<br>87	0.56<br>***<0.001***<br>87	0.24<br>***0.03***<br>87	0.22<br>***0.04***<br>87	0.10<br>*0.33*<br>87	-0.01<br>*0.89*<br>87	-0.38<br>***<0.001***<br>87
**Senescent CD4**	---	---	---	---	---	---	1.00	0.64<br>***<0.001***<br>89	0.33<br>***0.002***<br>89	0.36<br>***<0.001***<br>89	0.25<br>***0.02***<br>89	0.08<br>*0.45*<br>89	-0.37<br>***<0.001***<br>89
**Senescent CD8**	---	---	---	---	---	---	---	1.00	0.37<br>***<0.001***<br>89	0.37<br>***<0.001***<br>89	0.18<br>*0.08*<br>89	0.03<br>*0.77*<br>89	-0.51<br>***<0.001***<br>89

AAR = Age-Acceleration Residual, EEAA = Extrinsic Epigenetic Age Acceleration, PEAA = Phenotypic Epigenetic Age Acceleration, GEAA = Grim Epigenetic Age Acceleration, aaDNAmTL = age-adjusted DNA methylation-based estimate of telomere length, SN = HIV seronegative group

a: p values for pairwise correlations in italics, bold if <0.05

Table S8: Individual parameter estimates and *p* values from mixed effects models incorporating natural log-transformed absolute cell counts for T cell subsets for all five epigenetic measures, related to Table 4

		AAR		EEAA		PEAA		GEAA		aaDNAmTL	
**Co-variate**	**Category**	**Estimate (SE)[a], years**	***p value[b]***	**Estimate (SE), years**	***p value***	**Estimate (SE), years**	***p value***	**Estimate (SE), years**	***p value***	**Estimate (SE), relative units**	***p value***
Intercept	n/a	-5.6 (5.6)	*0.32*	8.6 (7.2)	*0.23*	3.1 (7.9)	*0.70*	-10.0 (3.2)	***0.002***	0.210 (0.257)	*0.42*
Study Visit	Visit A	-0.6 (0.6)	*0.32*	-0.7 (0.7)	*0.32*	-0.3 (0.8)	*0.71*	0.4 (0.3)	*0.11*	-0.002 (0.026)	*0.95*
	Visit B	0	.	0	.	0	.	0	.	0	.
HIV Serostatus Group[c]	SC	1.1 (0.7)	*0.13*	4.2 (0.9)	***<0.001***	5.4 (1.0)	***<0.001***	1.0 (0.5)	*0.05*	-0.299 (0.035)	***<0.001***
	SN	0	.	0	.	0	.	0	.	0	.
Study Visit*HIV Serostatus Group	SC*Visit A	0.4 (1.0)	*0.71*	-2.7 (1.2)	***0.03***	-2.7 (1.4)	***0.04***	0.4 (0.4)	*0.44*	0.179 (0.048)	***<0.001***
	SC*Visit B	0	.	0	.	0	.	0	.	0	.
	SN*Visit A	0	.	0	.	0	.	0	.	0	.
	SN*Visit B	0	.	0	.	0	.	0	.	0	.
CD4 T cells[d], ln cells/mm^3	n/a	1.6 (1.0)	*0.12*	3.5 (1.3)	***0.01***	3.4 (1.4)	***0.02***	1.3 (0.6)	***0.03***	-0.190 (0.048)	***<0.001***
CD8 T cells, ln cells/mm^3	n/a	0.1 (1.0)	*0.89*	-2.9 (1.3)	***0.03***	-1.7 (1.5)	*0.27*	0.6 (0.6)	*0.32*	0.119 (0.050)	***0.02***
Naïve CD4 T cells, ln cells/mm^3	n/a	-1.7 (0.6)	***0.01***	-3.6 (0.8)	***<0.001***	-3.4 (0.9)	***<0.001***	-0.4 (0.4)	*0.30*	0.129 (0.030)	***<0.001***
Activated CD8 T cells, ln cells/mm^3	n/a	-1.1 (0.4)	***0.01***	-0.8 (0.6)	*0.17*	-1.0 (0.6)	*0.10*	0.0 (0.2)	*0.90*	0.028 (0.021)	*0.18*
Senescent CD8 T cells, ln cells/mm^3	n/a	1.6 (0.4)	***<0.001***	2.0 (0.5)	***<0.001***	1.3 (0.6)	***0.02***	-0.3 (0.3)	*0.33*	-0.097 (0.019)	***<0.001***

AAR = Age-Acceleration Residual, EEAA = Extrinsic Epigenetic Age Acceleration, PEAA = Phenotypic Epigenetic Age Acceleration, GEAA = Grim Epigenetic Age Acceleration, aaDNAmTL = age-adjusted DNA methylation-based estimate of telomere length

a: parameter estimate and standard error (SE) from mixed models for each co-variate; degrees of freedom=168
b: Pr>t p values from mixed models for differences from zero for each co-variate, while holding all other co-variates fixed; p values in italics, bold if <0.05
c: HIV serostatus groups classified as SC (became HIV infected and seroconverted between Visits A and B) vs SN (persistently HIV uninfected and seronegative at Visits A and B)
d: absolute counts of T cell subsets as described in STAR Methods and Supplemental Table 13; all cell counts natural log-transformed (ln) for analyses

Table S9: Mean percentages of total T cells and T cell subsets within the live lymphocyte population of the SC and SN groups, at Visits A and B, related to Table 3

	**Visit A**[b], Mean % of live lymphocytes (SE)			**Visit B**[c], Mean % of live lymphocytes (SE)		
**T cell population**[a]	**SC** (n=92)	**SN** (n=90)	***p value***[d]	**SC** (n=92)	**SN** (n=93)	***p value***[d]
Total T cells	67.13% (0.84)	66.88% (1.02)	*0.849*	69.51% (0.82)	66.34% (1.25)	***0.036***
CD4 T cells	61.64% (0.95)	60.31% (1.00)	*0.333*	41.08% (1.33)	61.63% (1.06)	***< 0.001***
CD8 T cells	32.62% (0.84)	33.45% (0.91)	*0.500*	52.40% (1.29)	32.51% (0.98)	***< 0.001***
Naïve ($CD45RA^+CCR7^+$) CD4 T cells	15.39% (0.67)	16.12% (0.77)	*0.475*	11.18% (0.63)	15.83% (0.78)	***< 0.001***
Naïve ($CD45RA^+CCR7^+$) CD8 T cells	7.55% (0.38)	8.06% (0.40)	*0.362*	5.76% (0.36)	7.41% (0.34)	***0.001***
Activated ($HLA\text{-}DR^+CD38^+$) CD4 T cells	1.09% (0.04)	1.05% (0.05)	*0.596*	1.43% (0.06)	1.09% (0.05)	***< 0.001***
Activated ($HLA\text{-}DR^+CD38^+$) CD8 T cells	0.93% (0.06)	0.86% (0.07)	*0.523*	7.12% (0.53)	0.92% (0.11)	***< 0.001***
Senescent ($CD28^-CD57^+$) CD4 T cells	1.53% (0.18)	1.21% (0.13)	*0.150*	1.62% (0.18)	1.29% (0.14)	*0.148*
Senescent ($CD28^-CD57^+$) CD8 T cells	3.68% (0.26)	4.03% (0.35)	*0.417*	5.85% (0.38)	4.02% (0.39)	***0.001***

a: Total CD3+ T cells and T cell subsets determined by multicolor flow cytometry at the time of thawing of viable PBMC aliquots as described in the STAR Methods and Supplemental Table 13; percentages of total live lymphocytes calculated from live lymphocyte gate defined by forward vs. side scatter and Zombie Aqua viability dye. Flow cytometry data missing on some PBMC aliquots, but samples described here match those utilized in mixed models reported in Supplemental Table 10.
b: all participants HIV uninfected at Visit A, matched on age and hepatitis C status
c: SC recently HIV-infected, SN persistently HIV uninfected at matched time intervals at Visit B
d: p values are for comparison of SC vs. SN at each visit by t-tests; p values in italics, bold if <0.05

Table S10: Potential contribution of frequencies of T cells within the live lymphocyte population to epigenetic measures over time, using mixed effects models, related to Table 4

**Potential Contributors to Epigenetic Measures**	F value *(p value)*[a]				
	**AAR**	**EEAA**	**PEAA**	**GEAA**	**aaDNAmTL**
Study Visit, Visit A vs B	7.79 (**0.01**)	7.65 (**0.01**)	4.13 (**0.04**)	1.65 (0.20)	15.99 (**<0.001**)
HIV Serostatus Group, SC vs SN[b]	1.76 (0.19)	16.92 (**<0.001**)	18.49 (**<0.001**)	4.33 (**0.04**)	43.34 (**<0.001**)
Study Visit*HIV Serostatus Group	0.18 (0.19)	4.22 (**0.04**)	6.26 (**0.01**)	0.14 (0.70)	16.82 (**<0.001**)
CD4 T cells[c], In % of live lymphocytes	0.78 (0.38)	12.06 (**0.001**)	10.71 (**0.001**)	0.29 (0.59)	18.47 (**<0.001**)
CD8 T cells, In % of live lymphocytes	0.01 (0.92)	0.09 (0.76)	0.41 (0.52)	0.03 (0.87)	0.04 (0.84)
Naïve CD4 T cells, In % of live lymphocytes	6.58 (**0.01**)	42.02 (**<0.001**)	25.93 (**<0.001**)	2.14 (0.14)	18.64 (**<0.001**)
Activated CD8 T cells, In % of live lymphocytes	2.98 (0.09)	0.15 (0.69)	0.79 (0.38)	0.17 (0.68)	0.54 (0.47)
Senescent CD8 T cells, In % of live lymphocytes	10.98 (**0.001**)	7.51 (**0.01**)	2.13 (0.15)	1.31 (0.25)	19.58 (**<0.001**)

AAR = Age-Acceleration Residual, EEAA = Extrinsic Epigenetic Age Acceleration, PEAA = Phenotypic Epigenetic Age Acceleration, aaDNAmTL = age-adjusted DNA methylation-based estimate of telomere length

a: F values and Pr >F p values from mixed models incorporating all potential co-variates for all participants at both visits (n=382 out of 407 possible observations due to missing data for some co-variates) in a single model; p values in italics, bold if <0.05

b: HIV serostatus groups classified as SC (became HIV-infected and seroconverted between Visits A and B) vs SN (persistently HIV uninfected and seronegative at Visits A and B)

c: % of T cell subsets in live lymphocyte population as described in STAR Methods and Supplemental Table 13; all % natural log-transformed (ln) for analyses

Table S11: WGCNA Results, listing all CpGs in 18 Modules significantly associated with initial HIV infection in SC group, related to Table 5 (Excel file)

Table S12: Enrichment Analyses in Modules 1-18 for biological pathways containing genes with at least one CpG with kME ≥0.85 in WGCNA, related to Table 5 (Excel file)

Table S13: Multicolor flow cytometry panels for determination of total CD3 T cells, and CD4, CD8, naïve CD4 or CD8, activated CD4 or CD8, and senescent CD4 or CD8 T cell subsets, related to STAR Methods

	**Attune NxT Flow Cytometer Channel**							
	**BL-2**	**RL-1**	**RL-3**	**VL-1**	**VL-2**	**VL-3**	**YL-1**	**YL-3**
Antibody Label	*PerCP*	*AF647*	*APC-Cy7*	*V450*	*Zombie Aqua*	*BV605*	*PE*	*PE-Cy7*
Naïve and Senescent tube (antibody volume/tube)	CD3 (20 µL)	CCR7 (20 µL)	CD8 (5 µL)	CD4 (5 µL)	Zombie Aqua (100 µL)	CD57 (5 µL)	CD28 (20 µL)	CD45RA (5 µL)
Activated tube (antibody volume/tube)	CD3 (20 µL)	----	CD8 (5 µL)	CD4 (5 µL)	Zombie Aqua (100 µL)	HLA-DR (5 µL)	CD38 (20 µL)	----
Isotype tube (antibody volume/tube)	CD3 (20 µL)	IgG2a (5 µL)	CD8 (5 µL)	CD4 (5 µL)	Zombie Aqua (100 µL)	IgG2a (2.5 µL)	IgG1 (20 µL)	IgG1 (5 µL)

PerCP = Peridinin-Chlorophyll-Protein, AF647 = Alexa Fluor 647, APC-Cy7 = Allophycocyanin-Cyanine7 tandem, V450 = horizon V450, BV605 = Brilliant Violet 605, PE = Phycoerythrin, PE-Cy7 = Phycoerythrin-Cyanine7 tandem

Figure S1: Plasma HIV viral load (HIV VL) and EEAA, PEAA, and aaDNAmTL are correlated at the post-HIV infection visit in SC, related to Figure 1 and Table 4. The correlations between epigenetic measures and HIV VL at Visit B in SC participants (n=102) are shown for: A) Age Acceleration Residual (AAR), B) Extrinsic Epigenetic Age Acceleration (EEAA), C) Phenotypic Epigenetic Age Acceleration (PEAA), D) Grim Epigenetic Age Acceleration (GEAA), and E) age-adjusted DNA methylation-based estimate of telomere length (aaDNAmTL). HIV VL is plotted (x axis) on a log10 scale, epigenetic measures are plotted (y axis) on the native scale. Pearson correlation coefficients (rho) and *p* values are shown.

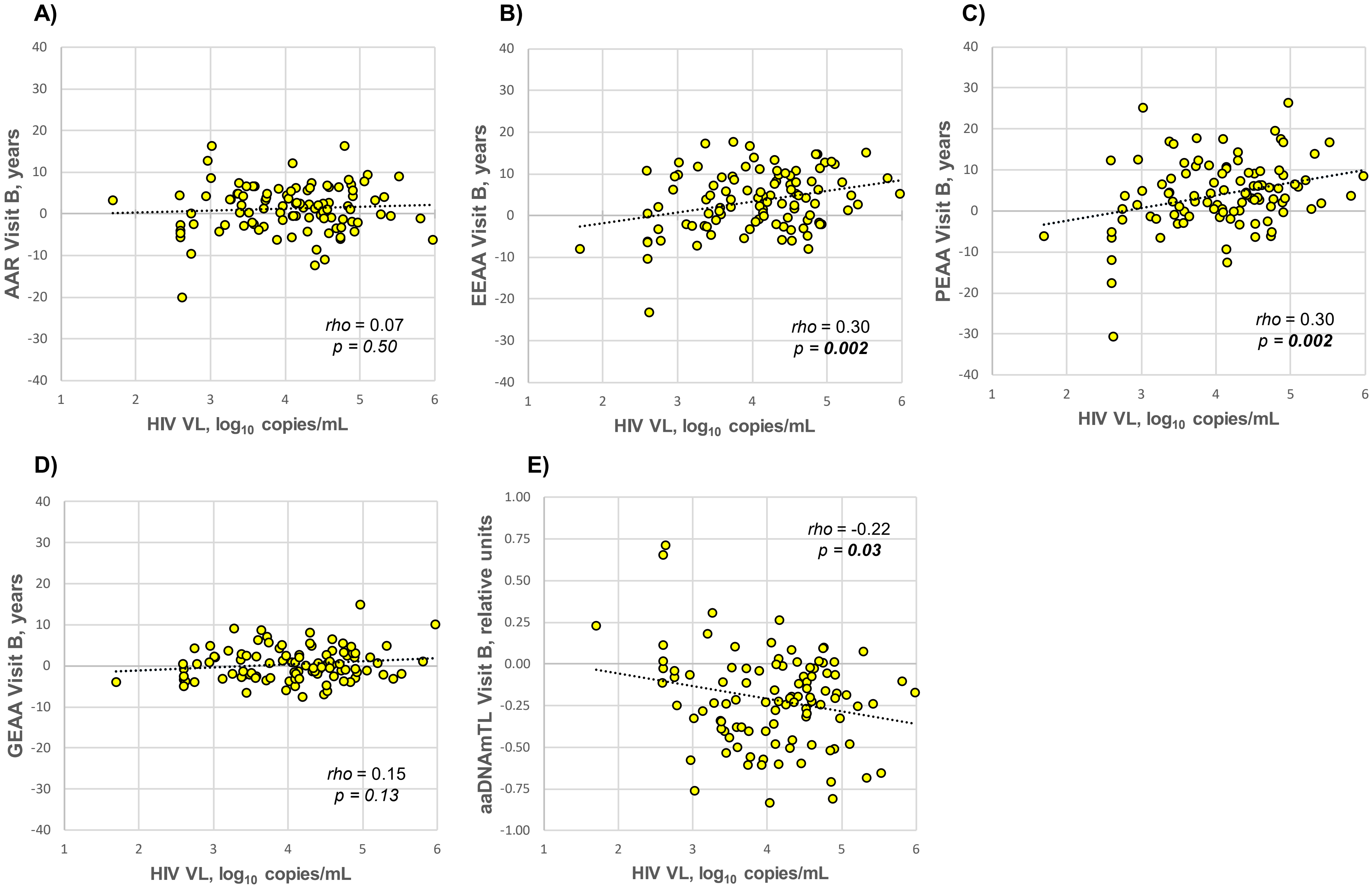

Supplement: Document S1. Tables S1–S13 and Figure S1 [file mmc1.pdf]
